# A Pharmacological Screening Approach for Discovery of Neuroprotective Compounds in Ischemic Stroke

**DOI:** 10.1371/journal.pone.0069233

**Published:** 2013-07-18

**Authors:** Simret Beraki, Lily Litrus, Liza Soriano, Marie Monbureau, Lillian K. To, Steven P. Braithwaite, Karoly Nikolich, Roman Urfer, Donna Oksenberg, Mehrdad Shamloo

**Affiliations:** 1 Behavioral and Functional Neuroscience Laboratory, Institute for Neuro-Innovation and Translational Neurosciences, School of Medicine, Stanford, California, United States of America; 2 BioSeek division of DiscoverX, South San Francisco, California, United States of America; 3 Pacific BioDevelopment Limited Liability Company, Emeryville, California, United States of America; 4 Circuit Therapeutics, Menlo Park, California, United States of America; 5 Selonterra Limited Liability Company, Belmont, California, United States of America; 6 Global Blood Therapeutics, South San Francisco, California, United States of America; St Michael’s Hospital, University of Toronto, Canada

## Abstract

With the availability and ease of small molecule production and design continuing to improve, robust, high-throughput methods for screening are increasingly necessary to find pharmacologically relevant compounds amongst the masses of potential candidates. Here, we demonstrate that a primary oxygen glucose deprivation assay in primary cortical neurons followed by secondary assays (i.e. post-treatment protocol in organotypic hippocampal slice cultures and cortical neurons) can be used as a robust screen to identify neuroprotective compounds with potential therapeutic efficacy. In our screen about 50% of the compounds in a library of pharmacologically active compounds displayed some degree of neuroprotective activity if tested in a pre-treatment toxicity assay but just a few of these compounds, including Carbenoxolone, remained active when tested in a post-treatment protocol. When further examined, Carbenoxolone also led to a significant reduction in infarction size and neuronal damage in the ischemic penumbra when administered six hours post middle cerebral artery occlusion in rats. Pharmacological testing of Carbenoxolone-related compounds, acting by inhibition of 11-β-hydroxysteroid dehydrogenase-1 (11β-HSD1), gave rise to similarly potent *in vivo* neuroprotection. This indicates that the increase of intracellular glucocorticoid levels mediated by 11β-HSD1 may be involved in the mechanism that exacerbates ischemic neuronal cell death, and inhibiting this enzyme could have potential therapeutic value for neuroprotective therapies in ischemic stroke and other neurodegenerative disorders associated with neuronal injury.

## Introduction

Stroke is the fourth leading cause of adult disability in the United States and a significant public health problem worldwide [1]. Neuroprotective therapies that can be administered after stroke to reduce further neuronal loss are, therefore, a critical area for research and drug development. Tissue plasminogen activator (tPA), currently the only approved therapy, must be administered within 3 hours of stroke onset and carries a risk of inducing cerebral hemorrhage (see review [2], [3]). Novel mechanisms and pharmacological agents are needed to treat patients who suffer a stroke in order to limit neuronal damage and improve clinical outcome. Here we report an approach to screen a library of pharmacologically active compounds in an *in vitro* model for ischemic injury using primary cortical neurons and hippocampal slices.

Understanding of the mechanisms underlying neuronal death has led to the proposal that several parallel cellular processes including excitotoxicity, ionic imbalance, oxidative stress, and apoptotic–like cell death contribute to delayed ischemic neuronal damage (see review [4], [5]). Despite numerous large clinical trials with compounds targeting these pathways at the individual level, none of these experimental treatments have been successful in generating lead therapeutics for ischemic stroke. This may further suggest that ischemic brain injury following stroke is mediated by activation of several of these complex signaling pathways, and targeting a selective signaling cascade would not be beneficial in protecting the tissue in this disorder. Therefore, approaches that can further define the mechanisms and relevance of pharmacological intervention are necessary to identify compounds of potential benefit.

In this study we used the oxygen glucose deprivation (OGD) model of ischemic neuronal death to identify neuroprotective compounds from a small library. With this approach, we identified Carbenoxolone, a compound known as a gap junction blocker (see review [6]) and modulator of 11-β-hydroxysteroid dehydrogenases [7], [8], as a neuroprotectant. This compound proved to be efficacious in an *in vivo* model of stroke and further delineation of its mechanism of action identified that inhibition of 11-β-hydroxysteroid dehydrogenase-1 (11β-HSD1) underlies, at least in part, its neuroprotective properties. The role of 11β-HSD1 is to modulate local levels of corticosteroids (reviewed in [9], [10]), acting as an oxoreductase to increase active glucocorticoid levels. Carbenoxolone’s neuroprotective properties were demonstrated in cultured hippocampal neurons [11], and 11β-HSD1 knockout mice are protected from age related decline in hippocampal function [12]. In addition, Carbenoxolone is neuroprotective when centrally [13] or peripherally [14] administered prior to ischemic injury.

The aim of this study was to discover development candidates by identifying neuroprotective compounds in primary cortical neurons and then confirm their activities in rodent models of stroke. After the initial screen, we focused our profiling on Carbenoxolone. Future efforts will extend our findings in further validating the importance of 11β-HSD1 in neuroprotection and prevention of functional loss in ischemic brain injury.

## Materials and Methods

### Ethics Statement

All experiments were in accordance with protocols approved by AGY’s Animal Care and Use Committee and were performed based on the National Institutes of Health Guide for the Care and Use of Laboratory Animals. Sufficient actions were considered for reducing pain or discomfort of subjects during the experiments.

### Animals and Reagents

All experimental procedures were approved by AGY’s Animal Care and Use Committee. Animal handling was performed in accordance with guidelines of National Institute of Health. Male Wistar rats were supplied by Harlan Laboratories (Harlan Inc., CA) at a body weight of 300–330 grams and approximately 9–10 weeks of age. The Library of Pharmacologically Active Compounds was purchased from Prestwick Chemical (The Prestwick Chemical Library, Illkirch, France) and all other chemicals were purchased from SigmaAldrich. BVT-2733 (3-chloro-2-methyl-N-(4-(2-(4-methylpiperazin-1-yl)-2-oxoethyl) thiazol-2-yl) benzenesulfonamide hydrochloride) was synthesized by a contract research organization.

### Hippocampal Slice Cultures and Primary Cortical Neuronal Cultures

Rat hippocampal cultures were generated using techniques for culturing brain slices originally described in Stoppini et al [15] with modifications in Cronberg et al [16]. Briefly, the hippocampi of male rats were dissected and immersed in ice-cold HBSS, cut into 250-µm-thick sections using a tissue chopper and plated, one slice per insert, onto Millicell culture inserts (0.4 µm Millicell-CM, 12 mm in diameter, Millipore Corp., Bedford, MA). Cultures were maintained in a humidified atmosphere at 35°C in a CO2 incubator (Thermo-Forma Scientific, Marietta, MA) for 3 weeks before experiments. The culture medium, with osmolarity 330 mosM, consisted of 50% MEM (Eagles with Earl’s balanced salt solution), 25% heat inactivated horse serum, 18% HBSS and 2% B27 and was supplemented with 4 mM l-glutamine and 50 units of penicillin–streptomycin/ml. d-glucose was added to a final concentration of 20 mM. B27 was omitted after the first week of culture. All substances were from Invitrogen, Carlsbad, CA, with the exception of d-glucose, which was from Sigma, St. Louis, MO. Rat primary cortical neurons were prepared from E17 embryos. The brain cortices were dissected and the neurons dissociated, digested, and plated as previously described [17], [18]. Three days later, 5-fluoro-2′-deoxyuridine (30µM) was added. Cells were maintained for 12–14 days in Neurobasal medium (Gibco) supplemented with B27 (Gibco) and 2 mM glutamine in a humidified atmosphere at 37°C with 5% CO_2_.

### Oxygen Glucose Deprivation (OGD)

Primary neuronal cultures were subjected to oxygen glucose deprivation for 120 minutes at 37°C. The cultures were placed in an anaerobic chamber (Forma Scientific) and incubated with a balanced salt solution (116 mM NaCl, 5.4 mM KCl, 1 mM NaH_2_PO4, 1.8 mm CaCl_2_, 26.2 mM NaHCO_3_, 0.01 mM glycine, pH = 7.4) lacking glucose and aerated with an anaerobic gas mix (85% N_2_/5%CO_2_/10% H_2_) to remove residual oxygen. Control cultures were kept in the original Neurobasal media and were submitted to the anaerobic conditions. At the end of the OGD insult, the cells were removed from the anaerobic chamber, the OGD media was replaced with Neurobasal media containing B27, and the cells were incubated for an additional 24 hours [16]. The compounds were present for 60 minutes prior to deprivation, during the 120 minute OGD, and for 24 hours post-OGD (pre-during-post) or only post hypoxic-hypoglycemic episode.

### Propidium Iodide (PI) Staining

Cell death was measured with Propidium Iodide (PI) staining as described in Cronberg et al [16] with slight modification. PI (1 µg/mL) was added to the culture medium 24 hours before OGD insult. Images were captured pre-OGD and 24 hours post-OGD using a fluorescent microscope and camera. ImageJ software (National Institutes of Health, Bethesda, Maryland) was used to measure fluorescence intensity from the images, representing PI uptake. For each image, the mean fluorescence intensity (MFI) was recorded for six random square areas within the area of interest. One background MFI value was recorded from a random square area outside of the area of interest, in the upper left corner of the image slice. The six values were averaged and the MFI of the background staining was subtracted from this average and this result was reported as the final MFI for each image.

### NMDA Toxicity

Primary neuronal cultures were exposed to 25 µM NMDA for 10 min at 37°C in a control salt solution (25 mM Tris, pH = 7.4, 120 mM NaCl, 5.4 mM KCl, 1.8 mM CaCl_2_, 15 mM D-glucose) containing 0.01 mM glycine [18], [19]. The exposure solution of the cells was then washed away and replaced by Neurobasal media containing B27 and the cells were placed in an incubator for 24 hours to recover. The test compounds were added to the neurons 2 hours prior to the NMDA addition and were also present during the NMDA insult and the recovery period or added only after the NMDA episode.

### Cell Viability

Adenosine Triphosphate (ATP) content was measured as an index for cell viability using Celltiter Glo (Promega, Madison, WI) according to the manufacturer’s instructions. Cells were seeded at 10,000 cells per well in a 96-well plate. This was determined to be within the linear range of the Celltiter Glo assay via titration of 0 to 50,000 cells per well prior to experimentation based on the manufacturer guidelines.

### Middle Cerebral Artery Occlusion

The transient middle cerebral artery occlusion (tMCAo) was performed in male Wistar rats according to Memezawa et al. [20] with some minor modifications. Briefly, a small incision was made in the common carotid artery and a nylon monofilament was inserted into the internal carotid artery through the common carotid artery. An occlusion time of 90 minutes was allowed in all rats subjected to tMCAo after which the filament was removed. The body temperature of rats subjected to tMCAo was maintained at 37±1°C for 6 hours after the occlusion.

### Measurement of Infarct Volume

Rats were subjected to 90 minutes of tMCAo and were decapitated after 24 h of reperfusion for determination of infarction volume. The isolated brains were quickly placed in cold saline for 20 minutes, sliced in seven coronal slices (2 mm thick), and stained in a 1.0% 2,3,5- triphenyltetrazolium chloride (TTC) solution in saline at 37°C for 30 minutes [21]. The same procedures were performed for sham-operated animals. The stained brain tissues were fixed in 10% formalin in phosphate-buffered saline. The images were captured using a CCD camera (Panasonic Corporation, Japan) and the unstained damaged areas were defined as infarcted tissue and were quantified using Image Pro Plus 4.1 software (Media Cybernetics, Silver Spring, MD).

### Data Analysis

All data analysis was performed using Graphpad Prism version 5 (Graphpad Software, San Diego, CA). The D’Agostino & Pearson omnibus normality test was utilized to determine Gaussian distribution. All normally distributed values are presented in the text as mean ± Standard Error of Mean (SEM) while non-Gaussian distributed values are reported as median (range). Values of p<0.05 were considered statistically significant. Testing for significant differences between two groups was performed using an unpaired Student’s t-test for values with Gaussian distribution and a Mann-Whitney U-test for values without Gaussian distribution. Differences between three or more treatment groups were analyzed using one-way Analysis of Variance (ANOVA) for Gaussian distributions and the Kruskal-Wallis test for values without Gaussian distribution. For post-hoc analysis, either the Dunnett’s Multiple Comparison Test or the Dunn’s Multiple Comparison Test was done when appropriate. Statistical tests used for each data set are indicated in the figure legends.

## Results

**Figure 1:**
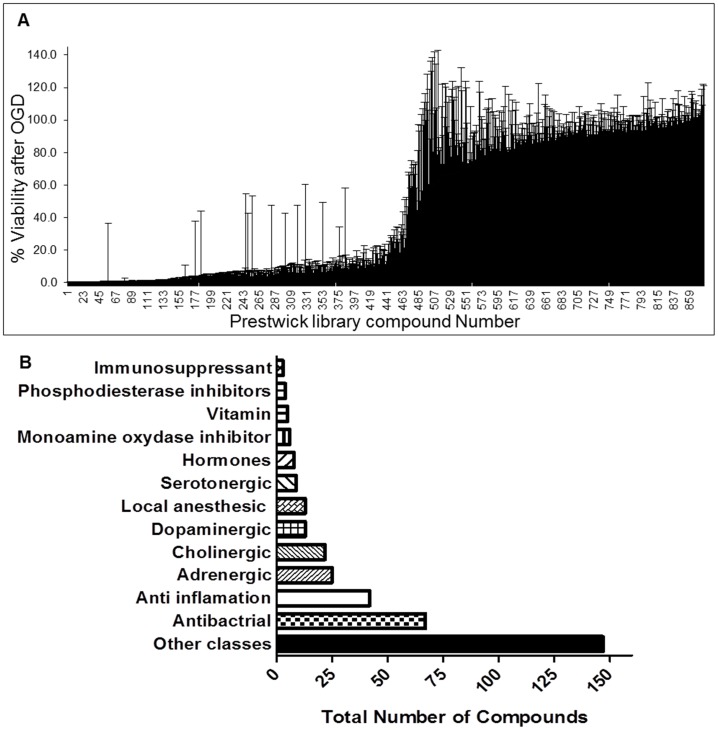
Neuroprotection and class of screened compounds. A library of pharmacologically active compounds was screened using an oxygen-glucose deprivation (OGD) assay with primary cortical neurons to identify neuroprotective compounds (**a**). At 24 hours post-OGD, approximately 50% of the 880 screened compounds showed neuroprotection at levels over 50% compared to controls (**a**). Compounds that showed protection represent an array of pharmacological classes including antibacterial, anti-inflammatory, anti-coagulant, and anti-hyperlipidemic compounds (**b**). The complete list of compounds tested and the degree of protection is displayed in Table S1.

We screened a library of pharmacologically active compounds using oxygen-glucose deprivation assays (OGD) with primary cortical neurons to identify potentially neuroprotective compounds for cerebral ischemia. In this initial screen of compounds at a concentration of 10µM, with the compound present pre-, during, and post-OGD (PDP), a remarkable 50% of the 880 screened compounds showed neuroprotection at a level of 50% of the positive control (Figure 1a). The complete list of the compounds tested and their level of neuroprotection is presented in the Table S1. Neuroprotective compounds belonged to a diverse set of pharmacological classes including antibacterial, anti-inflammatory, anti-coagulant, and antihyperlipidemic compounds (Figure 1b). We then selected 21 representative compounds from these classes (Figure 1b) and tested their neuroprotective activity in the OGD model when applied either PDP or only post OGD (Table 1). The majority of these compounds were neuroprotective when tested PDP while less than half displayed neuroprotective activity when tested post OGD (Table 1). We also tested these neuroprotective compounds in an NMDA-induced toxicity assay in which neuronal death is induced by 25µM NMDA. Sixty two percent of tested compounds showed neuroprotective activity with greater than 50% protection compared to the positive control when applied concurrently with NMDA and 45% of the tested compounds were neuroprotective when present only after the addition of NMDA to the cell cultures (Table 2). Out of these tested compounds, the following compounds displayed neuroprotective activity in all assays performed (PDP and Post treatment assays in both NMDA and OGD models): 1) Moxalactam disodium salt (Antibacterial), 2) Idoxuridine (Antiviral (anti-herpesvirus); Interacts with DNA, anticancer (glioma), radiation sensitizer), 3) Piperine (Antinematodal anti-inflammatory, hypotensive, chemopreventive, antioxidant, monoamine oxidase inhibitor), 4) Clofibric acid (Antilipidemic, cholesterol-lowering activity), 5) Meclofenoxate hydrochloride (Nootropic, Cholinergic agent), 6) Fipexide hydrochloride (Dopamine agonist, nootropic) 7) Catechin-(+,-) hydrate (Antioxidant, sulfated flavanoid pro-apoptotic, anti-proliferative, ameliorates cognitive impairement and neurodegeneration in an AD animal model).

**Table 1:** List of compounds displaying post-injury neuroprotective activity in the oxygen glucose deprivation (OGD) assay in cortical neurons.

		OGD 2 h		OGD 2 h		
Name		PDP (10µM)		Post (10µM)		Class
		% viability	(+/−)	% viability	(+/−)	
**1**	Moxalactam disodium salt	108.08	14.52	117.27	9.96	Antibacterial
**2**	Dapsone	148.05	22.26	62.70	72.24	Antibacterial (malaria, leprosy), neuroprotective against ischemia
**3**	Griseofulvin	122.59	12.85	37.72	45.80	Anti-inflammatory, anti-fungal; Disrupts microtubules
**4**	Sulfamonomethoxine	116.81	31.53	67.17	58.50	Sulphonamide, anti-infective; reduces myocardial reperfusion injury
**5**	Sulfaphenazole	94.86	7.50	31.34	31.86	Anti-infective, inhibits cytochrome P450
**6**	Idoxuridine	73.69	4.30	108.49	10.95	Antiviral (anti-herpesvirus); Interacts with DNA, anticancer (glioma), radiation sensitizer
**7**	Phenacetin	72.50	9.32	82.02	23.50	Anti-inflammatory, anti-analgesic, similar to acetaminophen
**8**	Fenspiride hydrochloride	88.50	5.29	60.88	27.82	Anti- inflammatory (pulmonary disease)
**9**	**Carbenoxolone disodium salt**	**89.34**	**1.93**	**86.21**	**3.15**	**Anti- inflammatory, antiulcer, HSD1, HSD2 inhibitor, GAP junction inhibitor**
**10**	Cyclophosphamide monohydrate	96.98	8.04	116.93	11.25	Immunosuppressant; Used to treat various types of cancer and autoimmune diseases; Neuroprotective in a gerbil model of focal ischemia
**11**	Azathioprine	53.23	20.36	58.96	25.42	Immunosuppressant; Used in Multiple Sclerosis and Crohn’s disease
**12**	Amiprilose hydrochloride	40.80	26.74	62.57	1.42	Immunosuppressant; Used to treat Rheumatoid Arthritis
**13**	Liothyronine	37.72	47.88	55.40	22.41	Thyroid hormone; Used to treat hypothyroidism
**14**	Chlorothiazide	50.98	32.54	62.89	1.33	Carbonic anhydrase inhibitor, antihypertensive
**15**	Acetazolamide	35.39	31.42	61.42	12.38	Carbonic anhydrase inhibitor, used for glaucoma, intracranial hyertenxion, and epileptic seizures
**16**	Methotrexate	84.02	4.53	86.64	9.00	Dihydrofolate reductase inhibitor; Used in treatment of cancer, autoimmune diseases
**17**	Amethopterin (R,S)	9.76	1.45	11.81	5.76	Dihydrofolate reductase inhibitor, similar to Methotrexate
**18**	Tranexamic acid	30.80	18.64	41.71	30.14	Antifibrinolyitic; Used in surgery and menstrual bleeding
**19**	Pilocarpine nitrate	40.83	33.67	30.94	38.94	M3 muscarinic receptor agonist, anti-glaucoma
**20**	Sulfinpyrazone	89.78	3.70	57.49	47.21	Uricosuric agent, antigout,anticoagulant, radical scavenger, MRP1 (multidrug resistant protein) inhibitor, anti-oxidant
**21**	Ganciclovir	85.99	9.60	54.94	22.27	Antiviral (anti-Cytomegalovirus, anti-herpesvirus); Used in liver transplantation
**22**	Azacytidine-5	76.64	6.52	57.50	9.21	Antineoplastic, demethylating agent
**23**	Piperine	74.58	7.65	63.59	12.60	Alkaloid in pepper, inhibits enzymes important for drugs metabolism, cognitive enhancing effects in ratsanti-inflammatory
**24**	Oxantel pamoate	72.97	7.65	15.79	12.95	Antinematodal for intestinal worms
**25**	Gemfibrozil	64.09	5.98	4.94	1.58	Antihyperlipidemic; Used together with statins as prevention for stroke, peroxisome proliferator-activated receptors α agonist
**26**	Clofibric acid	91.80	28.46	69.54	4.93	Antilipidemic; Cholesterol-lowering activity
**27**	Meclofenoxate hydrochloride	73.19	23.06	70.49	20.19	Nootropic, cholinergicagent, used to treat symptoms of senile dementia and Alzheimer disease
**28**	Fipexide hydrochloride	78.92	4.77	65.00	9.80	Dopamine agonist, nootropic
**29**	Catechin-(+,−) hydrate	84.66	11.82	29.65	29.30	Antioxidant, sulfated flavanoid pro-apoptotic, anti-proliferative, ameliorates cognitive impairement and neurodegeneration in an AD animal model

**Table 2:** List of compounds displaying post-injury neuroprotective activity in the NMDA-induced toxicity assay in cortical neurons.

		NMDA 25µM		NMDA 25µM		
Name		PDP (10µM)		Post (10µM)		Class
		% viability	(+/−)	% viability	(+/−)	
**1**	Moxalactam disodium salt	69.74	7.79	57.84	7.21	Antibacterial
**2**	Dapsone	85.50	2.67	55.23	31.42	Antibacterial (malaria, leprosy)
**3**	Griseofulvin	62.25	2.25	59.60	15.54	Anti-inflammatory, anti-fungal: Disrupts microtubules
**4**	Sulfamonomethoxine	93.95	3.67	83.75	11.35	Sulphonamide, anti-infective
**5**	Sulfaphenazole	79.67	3.43	74.80	6.28	Anti-infective
**6**	Idoxuridine	68.18	9.69	71.55	4.48	Antiviral (anti-herpesvirus); Interacts with DNA, anticancer (glioma), radiation sensitizer
**7**	Phenacetin	71.88	3.30	47.39	30.57	Anti-inflammatory, anti-analgesic
**8**	Fenspiride hydrochloride	67.68	10.22	58.05	16.51	Anti -inflammatory (pulmonary disease)
**9**	**Carbenoxolone disodium salt**	**41.98**	**3.89**	**9.63**	**1.75**	**Anti-inflammatory, antiulcer, HSD1, HSD2, GAP junction inhibitor**
**10**	Cyclophosphamide monohydrate	53.58	2.88	28.71	16.71	Immunosuppressant; Used to treat various types of cancer and autoimmune diseases
**11**	Azathioprine	54.68	5.79	43.29	24.88	Immunosuppressant; Used in Multiple Sclerosis, and Crohn’s disease
**12**	Amiprilose hydrochloride	70.97	7.25	54.48	36.45	Immunosuppressant; Used to treat Rheumatoid Arthritis
**13**	Liothyronine	7.21	0.73	35.50	39.79	Thyroid hormone; Used to treat hypothyroidism
**14**	Chlorothiazide	4.18	0.18	12.86	14.15	Carbonic anhydrase inhibitor, antihypertensive
**15**	Acetazolamide	13.82	4.21	40.36	28.13	Carbonic anhydrase inhibitor, sulfonamide (malaria)
**16**	Methotrexate	26.57	16.00	52.11	29.47	Dihydrofolate reductase inhibitor. Used in treatment of cancer, autoimmune diseases
**17**	Amethopterin (R,S)	45.62	20.20	47.95	38.17	Dihydrofolate reductase inhibitor, similar to Methotrexate
**18**	Tranexamic acid	28.47	2.36	34.49	4.75	Antifibrinolyitic; Used in surgery and menstrual bleeding
**19**	Pilocarpine nitrate	60.30	6.68	64.60	18.04	Cholinergic agonist
**20**	Sulfinpyrazone	75.49	8.18	74.93	9.63	Anticoagulants, radical scavenger, MRP1 (multidrug resistant protein) inhibitor, anti-oxidant, antigout
**21**	Ganciclovir	54.69	3.96	61.35	5.27	Antiviral (anti-Cytomegalovirus, anti-herpesvirus); Used in liver transplantation
**22**	Azacytidine-5	66.21	6.90	71.81	1.65	Antineoplastic, demethylating agent
**23**	Piperine	105.46	5.59	92.82	3.79	Antinematodal anti-inflammatory, hypotensive, chemopreventive, antioxidant, monoamine oxidase inhibitor
**24**	Oxantel pamoate	47.82	6.95	54.93	4.21	Antinematodal, cholinergic agent
**25**	Gemfibrozil	29.66	7.35	39.86	20.87	Antihyperlipidemic, Used together with statins as prevention for stroke, peroxisome proliferator-activated receptors α agonist
**26**	Clofibric acid	68.01	8.45	64.35	5.16	Antilipidemic, cholesterol-lowering activity
**27**	Meclofenoxate hydrochloride	70.16	1.38	61.97	2.26	Nootropic, cholinergic agent
**28**	Fipexide hydrochloride	85.74	6.05	61.08	3.49	Dopamine agonist, nootropic
**29**	Catechin-(+,−) hydrate	59.94	12.50	71.50	11.47	Antioxidant, sulfated flavanoid

**Figure 2:**
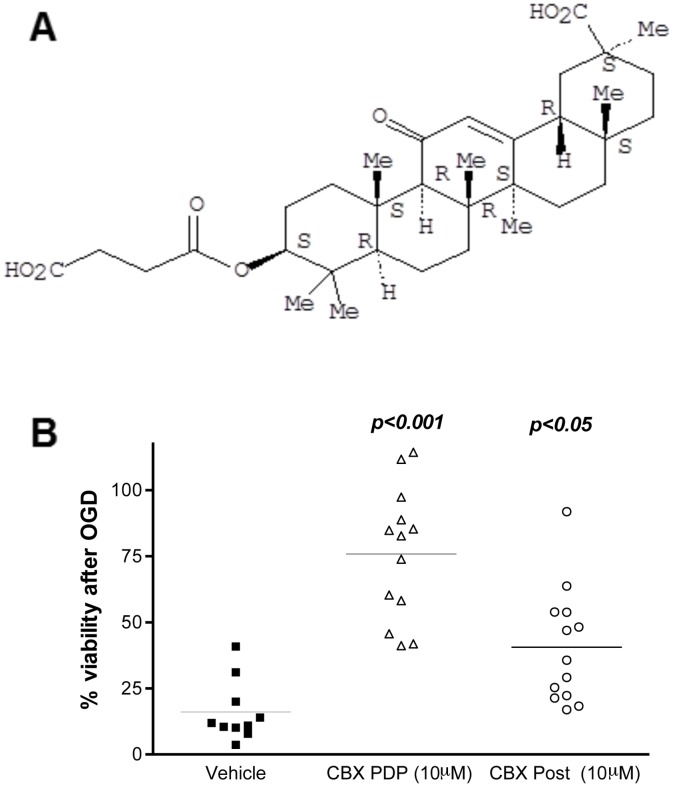
Molecular structure and neuroprotection of Carbenoxolone. Molecular structure of Carbenoxolone, a synthetic derivative (succinyl ester) of Glycyrrhetinic acid (constituent of licorice). Carbenoxolone is an inhibitor of 11β steroid dehydrogenase enzymes (HSD1 and HSD2) and gap junctions (**a**). Protection against OGD-induced neuronal damage by Carbenoxolone. Primary cortical neurons were subjected to 2 hours of OGD and neuronal damage was assayed using the Cell Titer Glo assay at 24 hours of recovery, in presence of vehicle, 10 µM Carbenoxolone pre-during-post (PDP) (***p<0.001 vs. Vehicle; n = 10–13), or exclusively post OGD (Post) (*p<0.05 vs. Vehicle; n = 10–13). Carbenoxolone demonstrated neuroprotective activity in both PDP and post treatment experiments (n = 10–13) (**b**). Data were assessed via one-way ANOVA and significant results of the Dunnett’s post-test are shown with lines representing mean.

**Figure 3:**
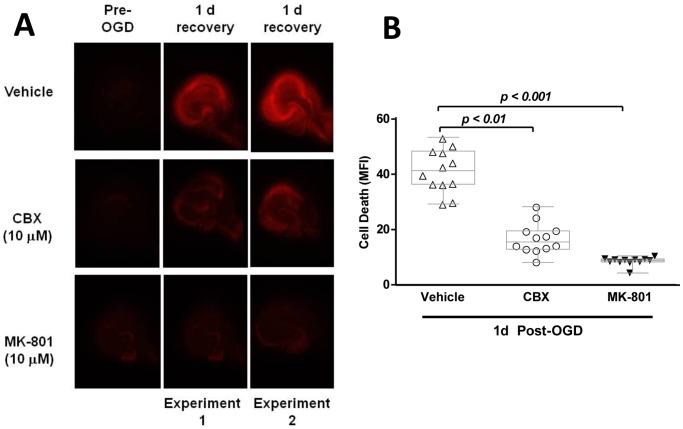
Carbenoxolone attenuates delayed OGD-induced hippocampal cell death. Hippocampal slice cultures were exposed to oxygen-glucose deprivation (OGD) and stained with Propidium iodide (PI). Photographs of the control and OGD slices at pre- and 24 hours post-OGD. A representative image is shown for each experiment (n = 4). MK-801 (Dizocilpine) was used as a positive control (**a**). The compounds were added 2 hours prior to the OGD insult. Mean fluorescence intensity (MFI) was measured 24 hours post-OGD. Both 10 µM Carbenoxolone (n = 12) (**p<0.01) and 10 µM MK-801 (n = 12) (***p<0.001) significantly reduced cell death compared to the vehicle group (n = 12) (**b**). Data were assessed using the Kruskal-Wallis test and significant results from Dunn’s Multiple Comparison test are displayed. Box plots represent median and quartiles and whiskers show minimum and maximum values.

To confirm the neuroprotective activity of one of these compounds, Carbenoxolone, primary cortical neurons were subjected to 2 hours of OGD and neuronal damage was assayed using the Cell Titer Glo assay at 24 hours of recovery. Carbenoxolone (10µM) demonstrated significant neuroprotective activity in both PDP (p<0.001 vs. Vehicle) and post-OGD (p<0.05 vs. Vehicle) (Figure 2b). The hippocampus is particularly vulnerable to ischemic damage. Therefore, Carbenoxolone’s neuroprotective activity was tested in OGD of organotypic hippocampal slices. This assay extends the investigation of Carbenoxolone’s neuroprotective activity to a model with an intact neuronal network. Cell death was assayed by Propidium Iodide staining prior to and one day after OGD (Figure 3). Slices treated with vehicle displayed high neurotoxicity subsequent to OGD throughout the hippocampus (Figure 3a). Treatment with 10µM Carbenoxolone significantly protected against hippocampal cell death (p<0.01 vs. Vehicle, Figure 3b). The degree of neuroprotection was similar to that detected by the NMDA receptor antagonist MK801 (p<0.001 vs. Vehicle), a proven neuroprotective agent that inhibits the calcium flux via the NMDA receptor in pretreatment models (Figure 3b).

**Figure 4:**
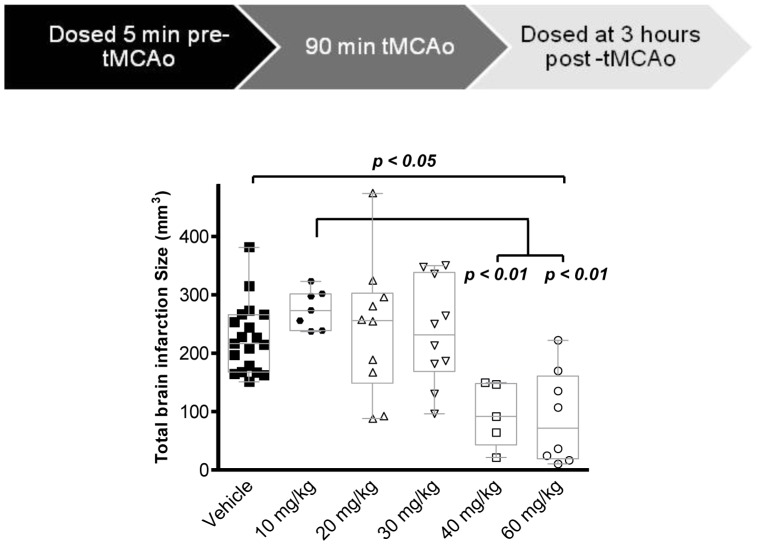
Treatment with Carbenoxolone attenuates ischemic brain injury. Animals were subjected to 90 minutes of tMCAo and were treated 5 minutes pre-tMCAo and 3 hours post-tMCAo with Carbenoxolone or vehicle (H_2_O) at the indicated total doses. Total infarction size was significantly decreased in tMCAo animals treated with 40 mg/kg (n = 5) (**p<0.01) and 60 mg/kg (n = 8) (**p<0.01) as compared to the 10 mg/kg treated group (n = 7) as well as in the 60 mg/kg (n = 8) (*p<0.05) group as compared to the vehicle group (n = 19). Data were assessed using the Kruskal-Wallis test and significant results from Dunn’s Multiple Comparison test are shown. Box plots represent median and quartiles and whiskers show minimum and maximum values.

**Figure 5:**
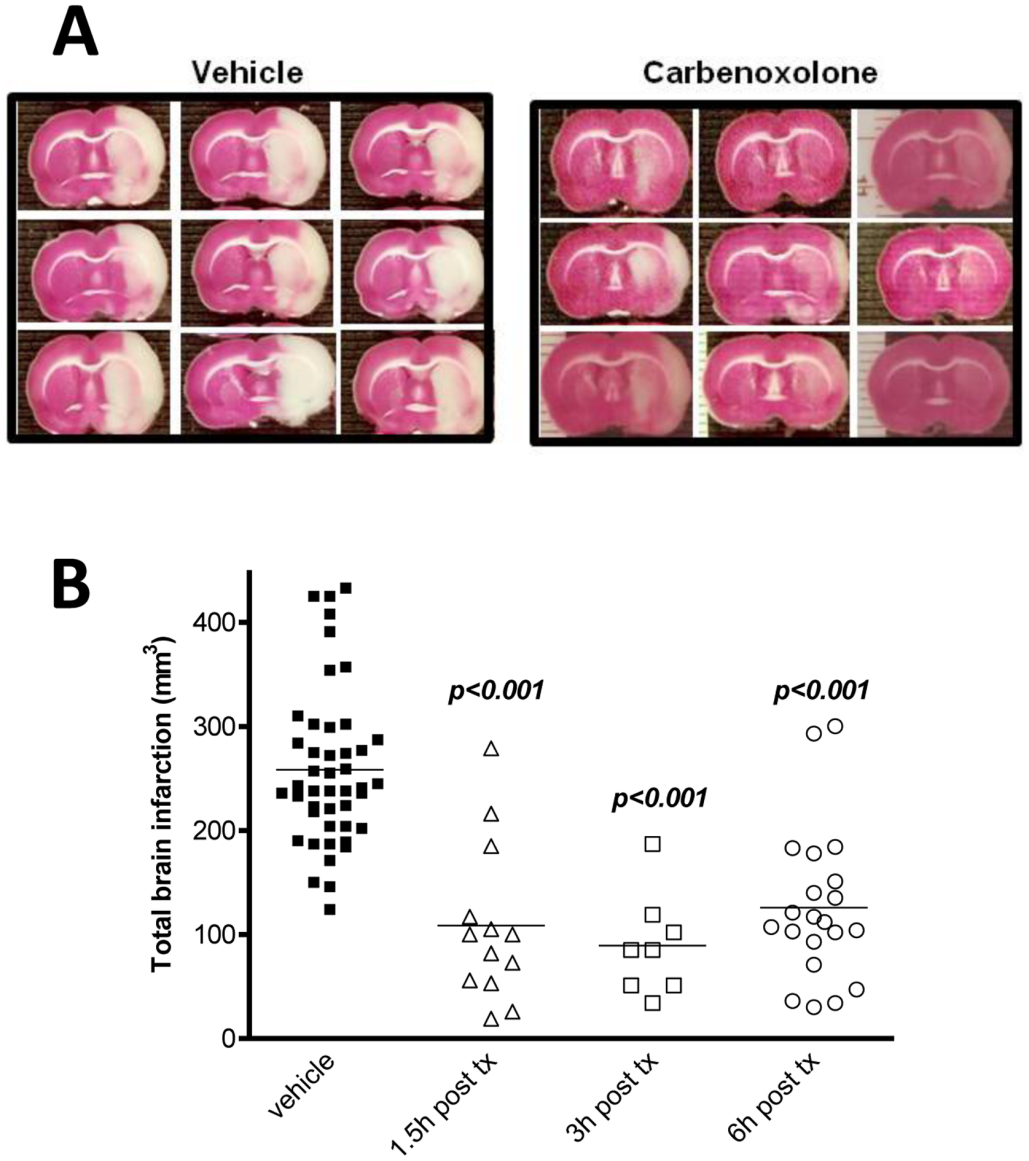
Post treatment in vivo efficacy of Carbenoxolone. Animals were subjected to 90 minutes of tMCAo treatment with 60 mg/kg total dose at 3 hours (30 mg/kg) and 6 hours (30 mg/kg) post-MCAo. Neuronal damage was quantified by TTC staining (n = 8–45), white (infarction), red (normal tissue) (**a**). Exploration of Carbenoxolone therapeutic window post-MCAo injury: Carbenonxolone was administered at a 60 mg/kg total dose (2×30 mg/kg) with a 3 hour interval with the first dose delivered at 1.5, 3, or 6 hours post-treatment (tx = treatment). The injuries in all the groups were quantified by TTC staining at 24 hours post injury (**b**). Data were assessed via one-way ANOVA and significant results of the Dunnett’s post-test and means are shown.

Transient middle cerebral artery occlusion (tMCAo) of rats was performed to assess the *in vivo* efficacy of Carbenoxolone in an animal model of stroke. To determine the most efficacious dose of carbenoxolone, the outcome after tMCAo was measured at doses of 10, 20, 30, 40, and 60 mg/kg (Figure 4). The treatment was administered at two timepoints with the first one given at 5 min pre-tMCAo and the second one at 3 hours post-tMCAo (Figure 4). Vehicle treated animals showed an infarct size of 225.2±13.5 mm^3^ (n = 19), measured 24 hours after the commencement of a 90 min tMCAo insult (Figure 4). Administration of Carbenoxolone at a total dose of 60 mg/kg (30 mg/kg 5 min prior to tMCAo; 30 mg/kg 3 hours post tMCAo) significantly reduced the brain infarct area (Figure 4, 90.3±28.4 mm^3^, p<0.05) compared to the vehicle group (Figure 4, 225.4±13.5 mm^3^) and compared to the 10 mg/kg treated group (Figure 4, 273.4 (237.4–323.4) mm^3^, p<0.01). The minimum significantly efficacious total dose was 40 mg/kg (20 mg/kg 5 min prior to tMCAo; 20 mg/kg 3 hours post tMCAo) with an infarct size of 91.8 (21.4–149.8) mm^3^ (Figure 4). In order to investigate the therapeutic window and whether Carbenoxolone retains its neuroprotective activity when treatment is initiated only after the neuronal injury, we administered Carbenoxolone at the same regimen (two doses of 30 mg/kg with a 3 hour interval for a total dose of 60 mg/kg) starting treatment at 1.5, 3, or 6 hours post-occlusion (Figure 5). This treatment regimen resulted in a reduction of the brain infarct area in 1.5 hours (108.5±21.1 mm^3^, p<0.001), 3 hours (89.3±17.2 mm^3^, p<0.001), and 6 hours (125.8±15.9 mm^3^, p<0.001) post treatment groups compared to vehicle (258.2±11.2 mm^3^) treated groups (Figure 5b).

**Figure 6:**
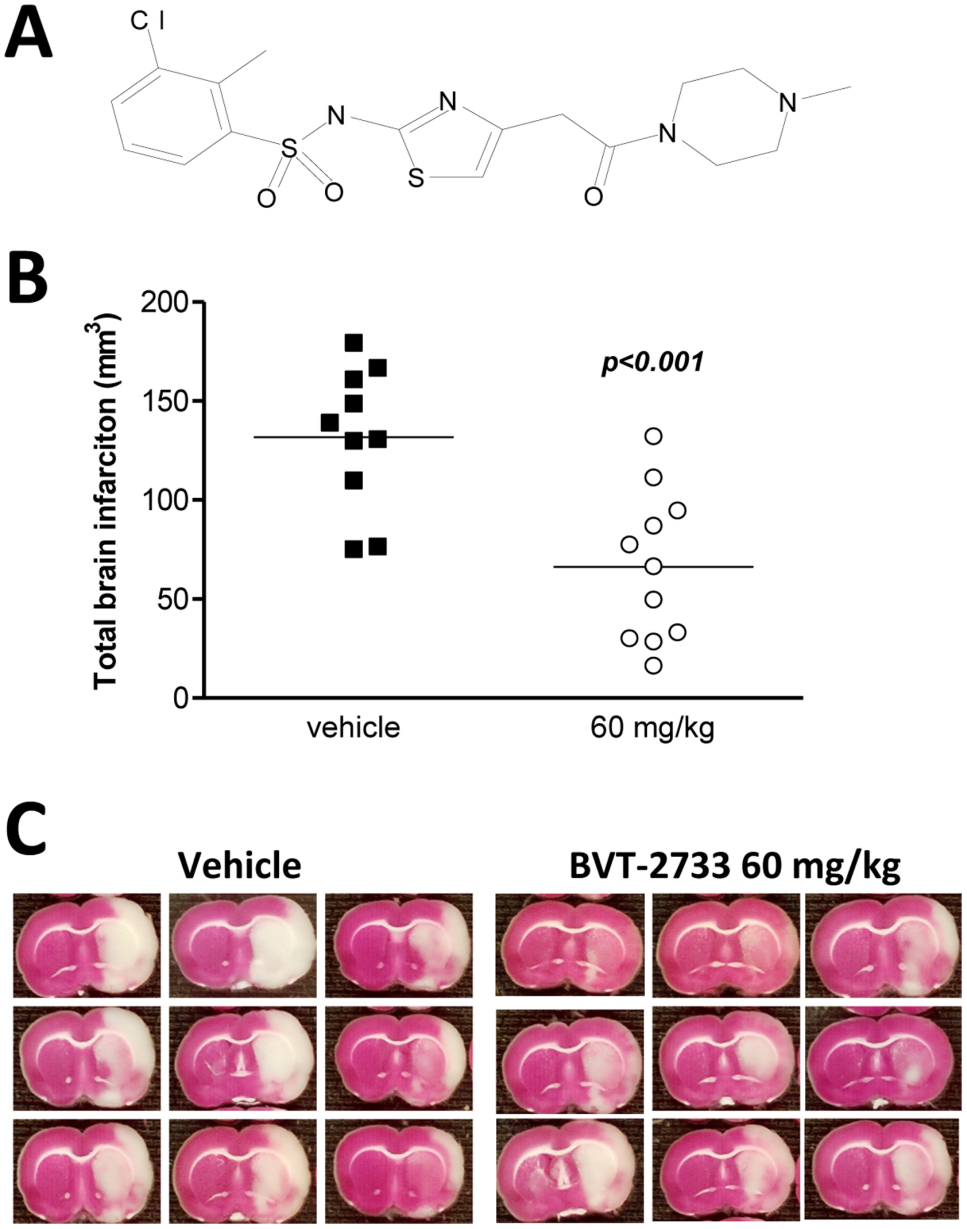
Molecular structure and neuroprotection of BVT-2733. Molecular structure of the specific 11β-HSD1 inhibitor, BVT-2733 (3-chloro-2-methyl-N-(4-(2-(4-methylpiperazin-1-yl)-2-oxoethyl) thiazol-2-yl) benzenesulfonamide hydrochloride) (**a**). Animals were subjected to 90 minutes of tMCAo and were treated with BVT-2733 30 mg/kg or vehicle (PEG 500 20%, DMSO 4%) at 3 hours and 7 hours post-reperfusion, for a total dosage of 60 mg/kg. Treatment with BVT-2733 (IP, intraperitoneal) (n = 10–11 in each treatment group) attenuated the ischemic brain injury (**b**). Data were assessed using an unpaired Student’s t-test. Scatter plots with mean values and significance is shown. Representative images of brain sections of treated animals: Neuronal damage was quantified by TTC staining; white indicates infarction and red staining indicates normal tissue (**c**).

In an independent study, we further explored the longer term functional recovery post stroke in Carbenoxolone treated animals. We found that all the animals treated with this neuroprotective dose of Carbenoxolone died within 7 days post-treatment. This finding demonstrates that a novel chemical entity could provide acute neuroprotective activity but could lack long-term functional improvement because of general toxicity. To investigate and potentially dissociate the mechanisms of Carbenoxolone’s neuroprotection and toxicity, respectively, we tested the hypothesis that inhibition of 11β-HSD1 mediates neuroprotection while inhibition of the 11β-HSD2 leads to general toxicity. Therefore, we tested the 11β-HSD1 specific inhibitor BVT-2733 in the tMCAo model in rats (Figure 6a). Treatment with 60 mg/kg BVT-2733 in two doses of 30 mg/kg, administered 3 and 7 hours post-reperfusion, resulted in a significant reduction in brain infarct volume (Figure 6b and 6c, vehicle 131.7±11.3 mm^3^, BVT-2733 66.2±11.4 mm^3^, p<0.001) compared to vehicle. *In vitro* analysis and long-term functional recovery testing of this compound has not yet been conducted; therefore we cannot conclude whether 11β-HSD1 specific inhibitor BVT-2733 is indeed less toxic than Carbenoxolone.

## Discussion

In the current study we screened a library of pharmacologically active compounds in order to identify novel therapeutic targets and compounds with neuroprotective activity. We identified 440 compounds with neuroprotective activity from over 12 therapeutic classes, including anti-inflammatory compounds and antibiotics. Our results showed that 50% of compounds screened in the pretreatment protocol displayed neuroprotective activity. We then made an educated selection of a subset of these compounds, and further confirmed compounds with a wide range of neuroprotective activity in both the NMDA toxicity assay as well as in OGD. Ten compounds (34%) showed neuroprotective activity in the post OGD assay and 13 compounds (45%) protected from NMDA excitotoxicity. We focused our efforts on Carbenoxolone, a synthetic derivative of Glycyrrhizinic acid, based on its described anti-inflammatory activity. Carbenoxolone was used clinically for treatment of oesophageal and ulcerative inflammation and has multiple biological effects that include the blocking of gap junctions [6], [22], [23] and non-specific inhibition of 11β-HSD enzymes [7]. Studies with cultured neurons have shown neuroprotection [24] and enhancement of NMDA induced cytotoxicity [25]. Additionally, Carbenoxolone was neuroprotective *in vivo* in a model of in utero hypoxia [26] and showed beneficial cognitive effects in clinical trials [27], [28]. However, the non-specific nature of Carbenoxolone’s mechanism led to clinical difficulties. In particular its inhibition of 11β-HSD2 was potentially responsible for hypertension and a syndrome of apparent mineralocorticoid excess associated with defects in the peripheral metabolism of cortisol (for review see [9]; [29]). The low blood-brain-barrier permeability of the compound [30] would necessitate large doses (40–60 mg/kg), which could in turn lead to a greater potential for peripheral side effects. In the present study, animals administered Carbenoxolone did not survive past 7 days post-treatment and long-term evaluation of neurological deficit was not possible. Therefore, despite acute beneficial *in vitro* and *in vivo* effects, it is unlikely that Carbenoxolone would become a viable drug for ischemic brain injury.

We therefore sought to understand the specific mechanism by which Carbenoxolone exhibits neuroprotection. Blockade of gap junctions is a viable mechanism for limiting neuronal damage in stroke as the ischemic insult and subsequent reperfusion can lead to aberrant neuronal firing between cells [31]. However, cortical neurons cultured under conditions with glial cell inhibition and a serum free media are unlikely to have significant gap junctional coupling and Carbenoxolone’s *in vitro* activity is unlikely to function through this mechanism, which is consistent with other studies [32]. Nevertheless, traffic of potentially harmful cytosolic messengers between ischemic cells and surrounding non-ischemic cells might cause an increase of post-stroke injury [33]. It is possible that minimizing gap junction permeability via a gap junction blocker before occluding the middle cerebral artery might reduce the infarct volume. Therefore, the possibility that Carbenoxolone acts via gap junction blockade to decrease infarction volume cannot be eliminated.

The other major functional mechanism of Carbenoxolone is the inhibition of 11β-HSD enzymes [34], [35] and, within the brain, 11β-HSD1 is by far the most prevalent isozyme (see review [36]). Inhibition of 11β-HSD2 is detrimental and is known to cause cortisol-dependent activation of the mineralocorticoid receptor with sodium retention resulting in hypertension [37]. We therefore studied the specific inhibition of the 11β-HSD1 isoform using the 11β-HSD1 specific inhibitor, BVT-2733. Indeed, BVT-2733 was capable of reducing brain infarct volumes in the rat tMCAo model, suggesting that Carbenoxolone’s neuroprotective properties are, at least partially, a result of 11β-HSD1 inhibition. 11β-HSD1 has an oxo-reductase activity capable of converting glucocorticoids from inactive to active forms at local sites of action [11], [38], [39]. It is expressed at high levels in CNS neurons ([40], [41], [42] See review [43]), as are corticosteroid receptors [8], suggesting that glucocorticoid regulation within the brain is functionally important. Circulating glucocorticoid levels are determined by the hypothalamic-pituitary-adrenal (HPA) axis, and pathological abnormalities in this axis have been linked to the risk of stroke [44]. Glucocorticoids have numerous functions in the brain’s response to stress, including regulation of synaptic plasticity [45] and mediating inflammatory responses. In stroke, inflammation is a key mediator of secondary neuronal damage [46]. Glucocorticoids are widely used as anti-inflammatory agents in the periphery; however, mounting evidence suggests that they can have pro-inflammatory roles in the CNS (reviewed in [47]). Our studies indicate that the localized modulation of glucocorticoid levels by 11β-HSD1 may be important in the secondary damage occurring in stroke. Therapeutic intervention to modulate glucocorticoid levels may therefore provide a novel mechanism for treating stroke. Furthermore, the neuroprotective activity of antibiotics reported in this study and in the literature [48] could also be explained by similar mechanistic pathways, inhibition of neuroinflammation, which is a secondary effect of this class of compounds [49].

The next phase of research will focus on exploring the potential use of Carbenoxolone-related compounds with 11β-HSD1 inhibition activity for neuroprotection. In particular, long-term neurological deficit evaluation needs to be completed in tMCAo rats subject to treatment to ensure that the neuroprotective activity seen in this study is the result of preventing injury rather that delaying injury. The current study measures infract volumes at 24 hours post-reperfusion. In addition, the hypothesis that inhibition of 11β-HSD2 causes the toxicity of Carbenoxolone, and therefore an exclusive 11β-HSD1 inhibitor such as BVT-2733 should provide neuroprotective benefits without toxicity, needs to be further explored in terms of functional recovery and long term protection. Once completed, this additional work will provide a substantial improvement to our understanding of the mechanism of neuroprotective activity of 11β-HSD1 inhibitors.

### Conclusions

**Figure 7:**
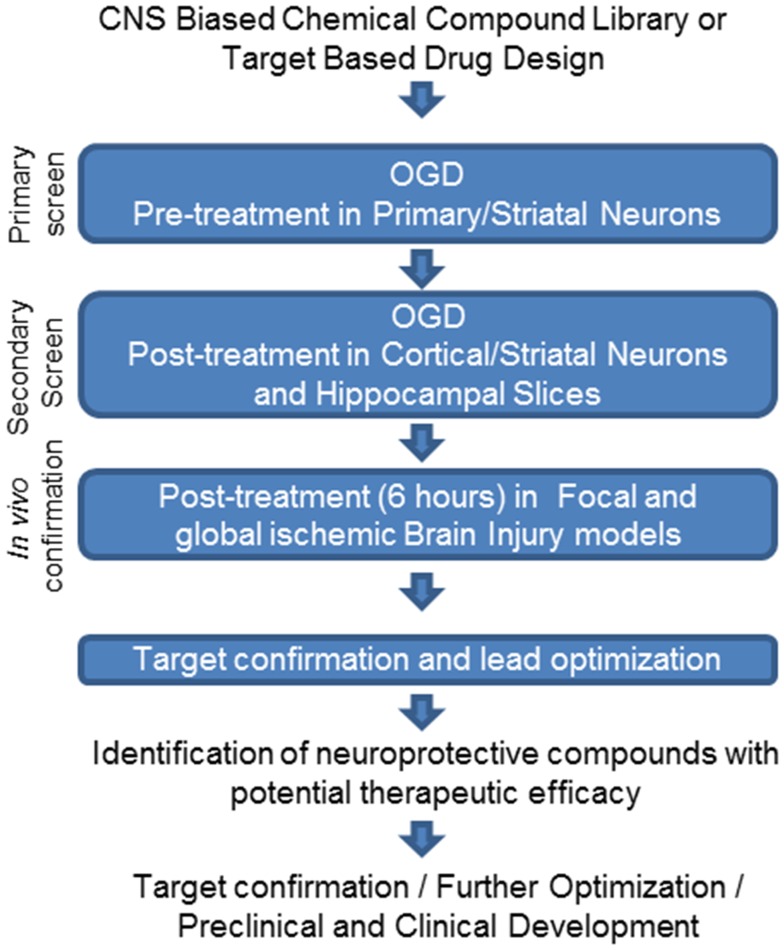
Profiling flow-chart to identify neuroprotective compounds with potential therapeutic efficacy.

We have demonstrated that OGD treatment in cortical neurons can be used as a primary screen to identify compounds with neuroprotective activity for ischemic stroke. Using this screening approach we have identified more than 400 compounds with neuroprotective activity in a pre-treatment protocol (Figure 7). However, just a few of these compounds displayed post-injury neuroprotective activity, which emphasizes the importance of applying post treatment protocols for screening and validation of neuroprotective compounds. We have shown that Carbenoxolone is a neuroprotective drug when given as late as 6 hours after the onset of the ischemic insult. However, the high doses used to achieve this neuroprotection can lead to toxicity. We showed that the neuroprotective activity of Carbenoxolone is mediated, at least in part, by inhibition of 11β-hydroxysteroid dehydrogenase type 1 (11β-HSD1). Our findings suggest that the increase of intracellular glucocorticoid levels mediated by 11β-HSD1 post brain injury may be a mechanism that exacerbates ischemic neuronal cell death, and inhibiting this enzyme could be used as a potential approach to neuroprotective therapies in ischemic stroke and other neurodegenerative disorders.

## Supporting Information

Table S1 Complete list of compounds tested and degree of neuroprotection. Neuroprotective compounds belonged to a diverse set of pharmacological classes including antibacterial, anti-inflammatory, anti-coagulant, and antihyperlipidemic compounds.
(DOCX)
