# Supplementary material for: A Pharmacological Screening Approach for Discovery of Neuroprotective Compounds in Ischemic Stroke
Source: PLoS One. 2013 Jul 18;8(7):e69233. doi: 10.1371/journal.pone.0069233 (PMC3715457; doi:10.1371/journal.pone.0069233)
Supplement: Table S1 — Complete list of compounds tested and degree of neuroprotection. Neuroprotective compounds belonged to a diverse set of pharmacological classes including antibacterial, anti-inflammatory, anti-coagulant, and antihyperlipidemic compounds. (DOCX) [file pone.0069233.s001.docx]

**Table S1.** Complete list of compounds tested and degree of neuroprotection.

|  | **OGD 2h** | |  | **OGD 2h** | |
| --- | --- | --- | --- | --- | --- |
| **Name** | **PDP (10µM)** | | **Name** | **PDP (10µM)** | |
|  | **% viability** | **(+/-)** |  | **% viability** | **(+/-)** |
| Pirenperone | 110.9 | 10.3 | Oxymetazoline hydrochloride | 10.8 | 9.6 |
| Ketanserin tartrate hydrate | 105.5 | 16.4 | Nitrarine dihydrochloride | 10.7 | 4.2 |
| Enalapril maleate | 103.8 | 5.4 | Droperidol | 10.7 | 11.4 |
| Piperine | 103.7 | 15.1 | Diazoxide | 10.6 | 11.9 |
| Ajmaline | 103.5 | 11.6 | Ethionamide | 10.5 | 0.7 |
| Methoxy-6-harmalan | 103.1 | 2.7 | Terbutaline hemisulfate | 10.3 | 3.9 |
| Nadolol | 102.9 | 0.0 | Carmustine | 10.3 | 7.6 |
| Guanethidine sulfate | 102.7 | 3.8 | Maprotiline hydrochloride | 10.1 | 12.6 |
| Orphenadrine hydrochloride | 101.6 | 2.3 | Ethosuximide | 10.1 | 7.6 |
| Doxazosin mesylate | 101.5 | 0.0 | Epiandrosterone | 9.9 | 12.6 |
| Tolmetin sodium salt dihydrate | 101.2 | 0.0 | Isoxicam | 9.5 | 1.6 |
| Calycanthine | 101.2 | 10.7 | Anthralin | 9.5 | 0.6 |
| Oxantel pamoate | 101.2 | 8.7 | Clorgyline hydrochloride | 9.4 | 11.9 |
| Minocycline hydrochloride | 100.8 | 6.0 | Piracetam | 9.3 | 10.1 |
| (+) -Levobunolol hydrochloride | 100.5 | 0.0 | Metoprolol-(+,-) (+)-tartrate salt | 9.3 | 1.6 |
| Pyrazinamide | 100.0 | 10.9 | Dantrolene sodium salt dihydrate | 9.3 | 2.6 |
| Phenacetin | 100.0 | 15.7 | Thioguanosine | 9.2 | 4.3 |
| Amprolium hydrochloride | 99.9 | 17.8 | Benfluorex hydrochloride | 9.1 | 3.5 |
| Moxalactam disodium salt | 99.6 | 13.5 | Anisomycin | 9.1 | 1.9 |
| Chlorphensin carbamate | 99.4 | 1.2 | Ofloxacin | 9.0 | 5.8 |
| Acetaminophen | 99.4 | 0.0 | Pempidine tartrate | 9.0 | 2.0 |
| Ifenprodil tartrate | 99.4 | 8.4 | Etoposide | 8.9 | 5.0 |
| Convolamine hydrochloride | 99.0 | 4.0 | Sulfameter | 8.9 | 11.6 |
| Mefexamide hydrochloride | 98.9 | 5.6 | Tetramisole hydrochloride | 8.8 | 0.1 |
| Cefsulodin sodium salt | 98.4 | 0.0 | Biotin | 8.8 | 5.0 |
| Dizocilpine maleate | 98.4 | 10.1 | Benzocaine | 8.7 | 14.1 |
| Guanabenz acetate | 98.4 | 4.7 | Midodrine hydrochloride | 8.6 | 9.9 |
| Allantoin | 98.1 | 16.3 | Furaltadone hydrochloride | 8.5 | 5.0 |
| Bendroflumethiazide | 98.1 | 4.3 | Puromycin dihydrochloride | 8.4 | 1.7 |
| Carbenoxolone disodium salt | 98.0 | 0.0 | Lycorine hydrochloride | 8.3 | 1.9 |
| Etodolac | 97.9 | 0.2 | Metampicillin sodium salt | 8.3 | 6.3 |
| (-) -Levobunolol hydrochloride | 97.6 | 7.5 | Erythromycin | 8.3 | 4.3 |
| Epirizole | 97.5 | 2.4 | Methapyrilene hydrochloride | 8.3 | 6.2 |
| Proglumide | 97.4 | 3.6 | Dicloxyacillin sodium salt | 8.3 | 0.4 |
| Haloperidol | 97.4 | 12.0 | Baclofen (R,S) | 8.3 | 7.8 |
| Yohimbine hydrochloride | 97.2 | 11.8 | Isopropamide iodide | 8.3 | 11.4 |
| Amiloride hydrochloride dihydrate | 97.1 | 20.8 | Busulfan | 8.3 | 5.4 |
| Telenzepine dihydrochloride | 97.0 | 4.2 | Sulfamethazine sodium salt | 8.2 | 2.6 |
| Mebeverine hydrochloride | 97.0 | 4.3 | Podophyllotoxin | 8.2 | 3.1 |
| Racecadotril | 96.9 | 13.1 | Glipizide | 8.2 | 2.4 |
| Naltrexone hydrochloride | 96.5 | 5.4 | Diphemanil methylsulfate | 8.2 | 4.9 |
| Fenspiride hydrochloride | 96.4 | 3.1 | Trimethobenzamide hydrochloride | 8.1 | 7.3 |
| Acetazolamide | 96.3 | 16.7 | Triamcinolone | 8.1 | 3.5 |
| Aminophylline | 96.3 | 9.8 | Triflusal | 8.1 | 2.2 |
| Mesna | 96.3 | 0.0 | Sisomicin sulfate | 8.1 | 3.1 |
| Kawain | 96.1 | 5.2 | Clonidine hydrochloride | 8.0 | 6.4 |
| Ginkgolide A | 96.1 | 5.5 | Cyclizine hydrochloride | 7.9 | 0.2 |
| Tetracycline hydrochloride | 96.0 | 5.6 | Heliotrine | 7.8 | 2.3 |
| Gemfibrozil | 95.6 | 3.5 | Sulfamerazine | 7.5 | 1.7 |
| Spironolactone | 95.5 | 11.1 | Isoniazid | 7.4 | 7.9 |
| Thiorphan | 95.4 | 9.7 | Testosterone propionate | 7.3 | 9.6 |
| Hydrastinine hydrochloride | 95.3 | 1.3 | Methazolamide | 7.2 | 51.0 |
| Pentolinium bitartrate | 95.2 | 0.3 | Chlortetracycline hydrochloride | 7.2 | 0.6 |
| Antipyrine | 95.2 | 2.9 | Flavoxate hydrochloride | 7.2 | 5.4 |
| Antipyrine, 4-hydroxy | 95.1 | 4.1 | Ricinine | 7.1 | 1.7 |
| Lomefloxacin hydrochloride | 95.0 | 7.0 | Vinpocetine | 7.1 | 9.3 |
| Amyleine hydrochloride | 94.9 | 0.1 | Sulfapyridine | 7.0 | 1.2 |
| Suprofen | 94.9 | 18.1 | Quipazine dimaleate salt | 7.0 | 9.3 |
| Dexamethasone acetate | 94.9 | 8.7 | Gelsemine | 7.0 | 3.8 |
| Fenfluramine hydrochloride | 94.9 | 4.2 | Ergocryptine-alpha | 6.9 | 27.3 |
| Griseofulvin | 94.9 | 3.0 | Acyclovir | 6.9 | 6.2 |
| Tinidazole | 94.9 | 7.2 | Theophylline monohydrate | 6.9 | 3.0 |
| Beclomethasone dipropionate | 94.9 | 0.0 | Oleandomycin phosphate | 6.8 | 3.1 |
| Methimazole | 94.8 | 2.0 | Mifepristone | 6.8 | 6.9 |
| Nifenazone | 94.8 | 7.3 | Amoxapine | 6.8 | 6.3 |
| Muramic acid, N-acetyl | 94.8 | 6.8 | Praziquantel | 6.7 | 5.6 |
| Harmaline hydrochloride dihydrate | 94.7 | 6.5 | Chelidonine monohydrate (+) | 6.7 | 4.7 |
| Phenindione | 94.6 | 16.3 | Cloxacillin sodium salt | 6.6 | 3.2 |
| Butacaine | 94.4 | 4.7 | Tetrahydrozoline hydrochloride | 6.6 | 8.5 |
| Moxisylyte hydrochoride | 94.3 | 3.8 | Cycloheximide | 6.6 | 0.6 |
| Cefoxitin sodium salt | 94.3 | 3.8 | 3-Hydroxymethyl-beta-carboline | 6.5 | 5.6 |
| Folic acid | 94.3 | 6.1 | Mevalonic-D, L acid lactone | 6.4 | 2.7 |
| Trichlormethiazide | 94.2 | 13.2 | Glibenclamide | 6.4 | 6.6 |
| Homatropine hydrobromide (R,S) | 94.2 | 2.1 | Propidium iodide | 6.4 | 1.7 |
| Catechin-(+,-) hydrate | 94.0 | 18.2 | Foliosidine | 6.4 | 1.0 |
| Cefadroxil | 93.9 | 5.3 | Succinylsulfathiazole | 6.3 | 0.7 |
| Lysergol | 93.8 | 8.8 | Dipyrone | 6.3 | 6.3 |
| Pepstatin A | 93.8 | 29.1 | beta- Belladonnine dichloroethylate | 6.3 | 0.2 |
| Cinchonidine | 93.7 | 13.3 | Alfuzocin hydrochloride | 6.3 | 5.0 |
| Securinine | 93.7 | 10.1 | Acacetin | 6.2 | 3.9 |
| Etidronic acid, disodium salt | 93.6 | 0.0 | Thioproperasine dimesylate | 6.1 | 4.5 |
| Clobetasol propionate | 93.6 | 2.2 | Josamycin | 6.1 | 4.4 |
| Dapsone | 93.5 | 10.8 | Carbinoxamine maleate salt | 6.0 | 43.2 |
| Equilin | 93.4 | 21.2 | Skimmianine | 6.0 | 0.5 |
| Mianserine hydrochloride | 93.4 | 6.3 | Carbimazole | 6.0 | 4.0 |
| Norfloxacin | 93.3 | #DIV/0! | Mafenide hydrochloride | 5.9 | 4.9 |
| Novobiocin sodium salt | 93.3 | 5.4 | Hydrochlorothiazide | 5.9 | 4.7 |
| Salbutamol | 93.3 | 5.6 | Sulfachloropyridazine | 5.8 | 4.7 |
| Sulfaphenazole | 93.3 | 9.6 | Molsidomine | 5.8 | 4.4 |
| Hydroflumethiazide | 93.1 | 4.1 | Resveratrol | 5.8 | 7.6 |
| Carbamazepine | 93.1 | 6.9 | Neomycin sulfate | 5.8 | 1.8 |
| Phenazopyridine hydrochloride | 93.0 | 9.0 | Eleagnine hydrochloride (R,S) | 5.7 | 5.8 |
| Liothyronine | 93.0 | 0.0 | Betaxolol hydrochloride | 5.7 | 7.2 |
| Nomifensine maleate | 93.0 | 9.6 | Bufexamac | 5.7 | 2.5 |
| Neostigmine bromide | 93.0 | 3.6 | Hydrocotarnine hydrobromide | 5.6 | 4.8 |
| Azaguanine-8 | 92.7 | 3.5 | Delcorine | 5.6 | 0.4 |
| Clofibric acid | 92.7 | 0.9 | Practolol | 5.6 | 6.8 |
| Cephalosporanic acid, 7-amino | 92.6 | 10.5 | Hycanthone | 5.6 | 2.8 |
| Glutethimide, para-amino | 92.5 | 10.6 | Pentoxifylline | 5.6 | 6.0 |
| Dilazep dihydrochloride | 92.4 | 10.2 | Sulfamethizole | 5.5 | 4.1 |
| Kynurenine, 3-hydroxy (R,S) | 92.4 | 0.6 | Dosulepin hydrochloride | 5.5 | 6.9 |
| Diperodon hydrochloride | 92.4 | 7.3 | Lovastatin | 5.5 | 8.7 |
| Pipenzolate bromide | 92.3 | 10.4 | Debrisoquin sulfate | 5.5 | 4.3 |
| Tranylcypromine hydrochloride | 92.2 | 6.9 | Chlorpropamide | 5.5 | 5.1 |
| Fenbufen | 92.2 | 7.3 | Zoxazolamine | 5.4 | 5.0 |
| Methionine sulfoximine (L) | 92.2 | 8.6 | Capsaicin | 5.4 | 5.2 |
| Lidocaine hydrochloride | 92.2 | 4.8 | Eucatropine hydrochloride | 5.3 | 55.1 |
| Doxycycline hydrochloride | 92.2 | 0.0 | Propafenone hydrochloride | 5.3 | 8.6 |
| Sulfacetamide sodic hydrate | 92.2 | 7.7 | Hydrocortisone base | 5.3 | 0.6 |
| Norethynodrel | 92.2 | 7.1 | Allopurinol | 5.2 | 3.6 |
| Strychnine | 92.2 | 3.1 | Levonordefrin | 5.2 | 0.8 |
| Phenylpropanolamine hydrochloride | 92.1 | 16.4 | Hippeastrine hydrobromide | 5.2 | 2.1 |
| Zomepirac sodium salt | 92.1 | 1.1 | Tiabendazole | 5.1 | 6.1 |
| Naringenine | 92.0 | 6.4 | Sulpiride | 5.1 | 4.4 |
| Ethynylestradiol 3-methyl ether | 91.9 | 5.1 | Chlorzoxazone | 5.1 | 5.7 |
| Lisinopril | 91.8 | 11.5 | Tetrazolone hydrochloride | 5.1 | 4.9 |
| Tranexamic acid | 91.8 | 5.8 | Bicuculline (+) | 5.1 | 0.5 |
| Alverine citrate salt | 91.6 | 2.5 | Mephentermine hemisulfate | 5.1 | 42.5 |
| Ketotifen fumarate | 91.6 | 7.6 | Bromopride | 5.1 | 0.9 |
| Oxalamine citrate salt | 91.6 | 23.9 | Phenelzine sulfate | 5.0 | 6.1 |
| Cromolyn disodium salt | 91.5 | 13.4 | Pramoxine hydrochloride | 5.0 | 5.0 |
| Naphazoline hydrochloride | 91.4 | 2.6 | Picrotoxinin | 5.0 | 5.9 |
| Amiprilose hydrochloride | 91.4 | 8.2 | Disopyramide | 5.0 | 6.4 |
| Sulfamonomethoxine | 91.4 | 10.8 | Medrysone | 4.9 | 7.5 |
| Ursolic acid | 91.4 | 15.8 | Trimetazidine dihydrochloride | 4.9 | 1.6 |
| Oxolinic acid | 91.4 | 1.0 | Loxapine succinate | 4.9 | 6.9 |
| Graveoline | 91.3 | 9.0 | Tremorine dihydrochloride | 4.8 | 6.3 |
| Trimethoprim | 91.3 | 5.1 | Trioxsalen | 4.8 | 6.0 |
| Thiamphenicol | 91.2 | 13.6 | Cinchonine | 4.8 | 4.7 |
| Mebhydroline 1,5-naphtalenedisulfonate | 91.2 | 11.6 | Khellin | 4.7 | 1.3 |
| Sulfadiazine | 91.1 | 3.2 | Nicergoline maleate | 4.7 | 3.0 |
| Isoxsuprine hydrochloride | 91.0 | 2.0 | Ajmalicine hydrochloride | 4.7 | 4.2 |
| Fenoterol hydrobromide | 91.0 | 12.7 | Cefoperazone dihydrate | 4.6 | 4.0 |
| Cinoxacin | 91.0 | 2.9 | Dihydrostreptomycin sulfate | 4.5 | 5.9 |
| Metaraminol bitartrate | 90.9 | 1.9 | Trimipramine maleate salt | 4.5 | 38.2 |
| Fillalbin | 90.8 | 9.6 | Chlorthalidone | 4.5 | 4.3 |
| Glycocholic acid | 90.8 | 3.1 | Norethindrone | 4.5 | 5.2 |
| Methylhydantoin-5-(L) | 90.8 | 14.5 | Lobelanidine hydrochloride | 4.5 | 6.0 |
| Naproxen | 90.7 | 3.2 | Betahistine mesylate | 4.4 | 5.4 |
| Clidinium bromide | 90.5 | 10.8 | Adenosine 5 -monophosphate monohydrate | 4.4 | 5.2 |
| Thalidomide | 90.5 | 2.5 | Berlambine | 4.4 | 5.3 |
| Benzydamine hydrochloride | 90.5 | 13.5 | Gentamicine sulfate | 4.3 | 3.0 |
| Mexiletine hydrochloride | 90.5 | 19.7 | Hydrastine hydrochloride | 4.3 | 3.8 |
| Ganciclovir | 90.5 | 5.6 | Atropine-N-oxide hydrochloride | 4.3 | 5.0 |
| Pyrilamine maleate | 90.5 | 8.3 | Melphalan | 4.2 | 0.9 |
| Alprenolol hydrochloride | 90.5 | 15.7 | Acetopromazine maleate salt | 4.1 | 2.6 |
| Meclofenamic acid sodium salt monohydrate | 90.4 | 2.3 | Tetrahydroalstonine | 4.1 | 3.6 |
| Probucol | 90.3 | 0.5 | Mefenamic acid | 4.0 | 1.5 |
| Enoxacin | 90.3 | 6.3 | Leflunomide | 4.0 | 5.0 |
| Methylergometrine maleate | 90.3 | 8.7 | Cefuroxime sodium salt | 4.0 | 5.2 |
| Buflomedil hydrochloride | 90.3 | 0.8 | Flumethasone | 4.0 | 0.5 |
| Mephenesin | 90.2 | 0.4 | Cefazolin sodium salt | 3.9 | 2.5 |
| Acebutolol hydrochloride | 90.2 | 12.6 | Dacarbazine | 3.9 | 2.4 |
| Metanephrine hydrochloride DL | 90.1 | 11.4 | Spectinomycin dihydrochloride | 3.9 | 43.7 |
| Lincomycin hydrochloride | 90.0 | 9.6 | Dibucaine | 3.9 | 3.7 |
| Gabapentin | 90.0 | 0.0 | Canavanine sulfate monohydrate (L,+) | 3.8 | 3.4 |
| Ethopropazine hydrochloride | 90.0 | 6.9 | Amrinone | 3.8 | 1.7 |
| Meticrane | 89.9 | 11.1 | Mometasone furoate | 3.8 | 4.1 |
| Ampyrone | 89.8 | 1.2 | Piromidic acid | 3.8 | 4.0 |
| Triamterene | 89.8 | 7.5 | Rifampicin | 3.8 | 4.7 |
| Benzthiazide | 89.8 | 15.4 | Omeprazole | 3.8 | 3.2 |
| Cephapirin sodium salt | 89.8 | 0.0 | Dichlorphenamide | 3.8 | 2.3 |
| Nafronyl oxalate | 89.8 | 3.7 | (R) -Naproxen sodium salt | 3.7 | 1.9 |
| Guaifenesin | 89.7 | 13.6 | Methotrimeprazine maleat salt | 3.7 | 1.7 |
| Vincamine | 89.6 | 14.6 | Theobromine | 3.7 | 2.0 |
| Bupivacaine hydrochloride | 89.6 | 14.7 | Mesoridazine besylate | 3.7 | 0.8 |
| Sulfinpyrazone | 89.5 | 9.9 | Retrorsine | 3.7 | 3.6 |
| Nafcillin sodium salt monohydrate | 89.4 | 7.3 | Coniine hydrochloride (D,L) | 3.7 | 4.0 |
| Cyanocobalamin | 89.4 | 5.0 | Levocarnitine | 3.6 | 2.7 |
| Nefopam hydrochloride | 89.3 | 4.5 | Benzbromarone | 3.5 | 4.4 |
| Paromomycin sulfate | 89.1 | 13.1 | Nadide | 3.5 | 4.0 |
| Bergenin monohydrate | 89.1 | 12.2 | Tomatidine | 3.5 | 3.9 |
| Hexamethonium dibromide dihydrate | 89.1 | 19.3 | Progesterone | 3.5 | 4.2 |
| Cefmetazole sodium salt | 89.1 | 7.2 | Carbenicillin disodium salt | 3.5 | 1.2 |
| Spiperone | 89.0 | 4.9 | Norcyclobenzaprine | 3.4 | 4.2 |
| Prilocaine hydrochloride | 89.0 | 2.6 | Ethacrynic acid | 3.4 | 2.7 |
| Ambroxol hydrochloride | 89.0 | 2.9 | Iohexol | 3.4 | 5.2 |
| Tiapride hydrochloride | 89.0 | 9.7 | Propylthiouracil | 3.4 | 3.3 |
| Chlorothiazide | 89.0 | 9.6 | Parthenolide | 3.4 | 0.4 |
| Cefotaxime sodium salt | 88.9 | 15.7 | Isopyrin hydrochloride | 3.4 | 50.0 |
| Flumequine | 88.9 | 2.1 | Xylometazoline hydrochloride | 3.4 | 3.4 |
| Adiphenine hydrochloride | 88.8 | 2.2 | (cis-) Nanophine | 3.3 | 0.4 |
| Cortisone | 88.6 | 16.1 | Hesperidin | 3.3 | 3.8 |
| Metrizamide | 88.6 | 3.7 | Scopolamine hydrochloride | 3.3 | 3.0 |
| Clemizole hydrochloride | 88.4 | 4.9 | Vigabatrin | 3.3 | 4.2 |
| Clopamide | 88.3 | 10.7 | Chloropyramine hydrochloride | 3.3 | 39.6 |
| Eburnamonine (-) | 88.3 | 7.6 | Boldine | 3.3 | 3.6 |
| Indomethacin | 88.3 | 10.2 | Sulfaguanidine | 3.3 | 1.3 |
| Cyclophosphamide monohydrate | 88.2 | 6.3 | Pyrithyldione | 3.2 | 51.4 |
| Trichlorfon | 88.2 | 1.7 | Reserpine | 3.2 | 5.9 |
| Ampicillin sodium salt | 88.1 | 15.5 | Xylazine | 3.2 | 4.3 |
| Gabazine | 87.9 | 4.7 | Homochlorcyclizine dihydrochloride | 3.2 | 1.8 |
| Procyclidine hydrochloride | 87.9 | 2.5 | Gramine, 7-benzyloxy | 3.2 | 4.1 |
| Etofylline | 87.9 | 7.8 | Ipratropium bromide | 3.1 | 2.7 |
| Tetrazoline hydrochloride | 87.8 | 9.7 | Viloxazine hydrochloride | 3.1 | 2.2 |
| Sulfamethoxazole | 87.8 | 5.4 | Trimethadione | 3.1 | 3.7 |
| Piperacillin sodium salt | 87.8 | 4.0 | Hecogenin | 3.1 | 1.2 |
| Ranitidine hydrochloride | 87.7 | 8.4 | Tolnaftate | 3.1 | 1.8 |
| Azacytidine-5 | 87.7 | 4.0 | Atractyloside potassium salt | 3.0 | 1.4 |
| Benoxinate hydrochloride | 87.5 | 2.4 | Seneciphylline | 3.0 | 3.1 |
| Chloramphenicol | 87.4 | 16.4 | Gramine | 3.0 | 2.5 |
| Pilocarpine nitrate | 87.3 | 4.2 | Methacholine chloride | 3.0 | 3.4 |
| Azlocillin sodium salt | 87.3 | 12.4 | 6-Furfurylaminopurine | 3.0 | 4.2 |
| Cephalexin monohydrate | 87.3 | 5.8 | Mycophenolic acid | 3.0 | 2.1 |
| Piroxicam | 87.2 | 1.5 | Chlorpromazine hydrochloride | 3.0 | 4.0 |
| Fluvastatin sodium salt | 87.1 | 0.5 | Arcaine sulfate | 2.9 | 2.3 |
| Domperidone | 87.1 | 8.2 | Sulfadimethoxine | 2.9 | 3.5 |
| Prednisolone | 87.1 | 18.3 | Napelline | 2.9 | 2.0 |
| Bumetanide | 87.0 | 18.4 | Monocrotaline | 2.9 | 2.9 |
| Terazosin hydrochloride | 86.8 | 9.6 | Lobeline alpha (-) hydrochoride | 2.8 | 3.7 |
| Carbetapentane citrate | 86.8 | 19.5 | Ascorbic acid | 2.8 | 4.0 |
| Indoprofen | 86.7 | 2.3 | Thiocolchicoside | 2.8 | 3.2 |
| Metoclopramide monohydrochloride | 86.7 | 4.9 | Deltaline | 2.8 | 3.0 |
| Captopril | 86.6 | 20.1 | Epivincamine | 2.8 | 3.3 |
| Troleandomycin | 86.5 | 15.6 | Lithocholic acid | 2.8 | 3.5 |
| Fluocinonide | 86.5 | 0.8 | Laudanosine (R,S) | 2.8 | 1.5 |
| Norgestrel-(-)-D | 86.4 | 9.1 | Myosmine | 2.7 | 2.6 |
| Methoxamine hydrochloride | 86.4 | 21.9 | Gliclazide | 2.7 | 2.5 |
| Isocorydine (+) | 86.4 | 9.4 | Salsolinol hydrobromide | 2.7 | 2.0 |
| Neamine | 86.3 | 29.0 | Ketoconazole | 2.7 | 3.0 |
| Cotinine (-) | 85.9 | 23.9 | Hexestrol | 2.7 | 3.8 |
| Phentolamine hydrochloride | 85.9 | 10.1 | Ribostamycin sulfate salt | 2.6 | 3.0 |
| Tadjakonine | 85.7 | 1.2 | Cephaeline dihydrochloride heptahydrate | 2.6 | 1.7 |
| Methenamine | 85.6 | 14.6 | Probenecid | 2.6 | 3.5 |
| Carisoprodol | 85.6 | 3.1 | Clofazimine | 2.5 | 2.6 |
| Midecamycin | 85.6 | 6.1 | Fluoxetine hydrochloride | 2.5 | 3.5 |
| Ketoprofen | 85.6 | 5.7 | Isoconazole | 2.5 | 3.1 |
| Oxytetracycline dihydrate | 85.4 | 13.0 | Kanamycin A sulfate | 2.5 | 2.4 |
| Methyl-6-thiouracil | 85.3 | 36.9 | Zaprinast | 2.5 | 3.0 |
| Chlorogenic acid | 85.3 | 1.0 | Trolox | 2.5 | 2.3 |
| Ethambutol dihydrochloride | 85.1 | 19.3 | Ticlopidine hydrochloride | 2.5 | 2.8 |
| Sulfathiazole | 84.9 | 6.5 | Flunisolide | 2.4 | 2.5 |
| Azacyclonol | 84.9 | 11.3 | Acenocoumarol | 2.4 | 1.2 |
| Heptaminol hydrochloride | 84.9 | 11.4 | Mepenzolate bromide | 2.4 | 1.9 |
| Aminopurine, 6-benzyl | 84.4 | 3.7 | Corynanthine hydrochloride | 2.4 | 3.1 |
| Antazoline hydrochloride | 84.4 | 13.6 | Bupropion hydrochloride | 2.4 | 2.0 |
| Fenoprofen calcium salt dihydrate | 84.3 | 1.0 | Conessine | 2.3 | 2.9 |
| Austricine | 84.3 | 13.2 | Tacrine hydrochloride hydrate | 2.3 | 2.7 |
| Zimelidine dihydrochloride monohydrate | 84.2 | 6.5 | pinacidil | 2.3 | 1.6 |
| Diphenylpyraline hydrochloride | 84.2 | 8.3 | Pregnenolone | 2.3 | 2.6 |
| Foscarnet | 84.2 | 26.1 | Dihydroergotoxine mesylate | 2.3 | 1.8 |
| Butamben | 84.2 | 18.9 | Dropropizine (R,S) | 2.3 | 1.6 |
| Buspirone hydrochloride | 84.1 | 6.2 | Hexetidine | 2.2 | 3.3 |
| Iproniazide phosphate | 83.9 | 13.6 | Evoxine | 2.2 | 1.7 |
| Bambuterol hydrochloride | 83.9 | 18.0 | Furazolidone | 2.2 | 2.7 |
| Idoxuridine | 83.8 | 3.8 | Halcinonide | 2.2 | 2.9 |
| Dextromethorphan hydrobromide monohydrate | 83.6 | 2.6 | Emetine dihydrochloride | 2.1 | 2.7 |
| Fipexide hydrochloride | 83.6 | 10.7 | Yohimbinic acid monohydrate | 2.1 | 2.2 |
| Metolazone | 83.6 | 6.8 | Atropine sulfate monohydrate | 2.1 | 2.7 |
| Levamisole hydrochloride | 83.4 | 4.4 | Terconazole | 2.1 | 1.7 |
| (d,l)-Tetrahydroberberine | 83.4 | 14.7 | Deferoxamine mesylate | 2.1 | 42.1 |
| Dobutamine hydrochloride | 83.4 | 11.5 | Amikacin | 2.0 | 2.0 |
| Cetirizine dihydrochloride | 82.7 | 2.1 | Pentamidine isethionate | 2.0 | 1.7 |
| Clebopride maleate | 82.6 | 8.0 | Tolazamide | 2.0 | 1.8 |
| Diprophylline | 82.5 | 8.9 | Metformin hydrochloride | 2.0 | 1.5 |
| Carcinine hydrochloride | 82.5 | 4.8 | Pentylenetetrazole | 2.0 | 1.0 |
| Tropicamide | 82.4 | 15.0 | Clomipramine hydrochloride | 2.0 | 1.7 |
| Trihexyphenidyl-D,L Hydrochloride | 82.4 | 3.7 | Thiamine hydrochloride | 1.9 | 2.3 |
| Meclofenoxate hydrochloride | 82.3 | 6.1 | Sulfamethoxypyridazine | 1.9 | 35.8 |
| Bromocryptine mesylate | 82.1 | 28.9 | Propantheline bromide | 1.9 | 1.0 |
| Tolazoline hydrochloride | 82.1 | 23.9 | Acetohydroxamic acid | 1.9 | 1.7 |
| Chenodiol | 82.1 | 0.1 | Thiourea, 1-phenyl-3-(2-thiazolyl)-2- | 1.9 | 2.3 |
| Dyclonine hydrochloride | 81.9 | 8.7 | Galanthamine hydrobromide | 1.8 | 2.2 |
| Minaprine dihydrochloride | 81.7 | 9.6 | Imipenem | 1.8 | 1.5 |
| Tetrahydroxy-1,4-quinone monohydrate | 81.5 | 30.7 | Glafenine hydrochloride | 1.7 | 0.3 |
| Pheniramine maleate | 81.4 | 15.9 | Etanidazole | 1.7 | 1.3 |
| Phthalylsulfathiazole | 81.2 | 24.4 | Flurandrenolide | 1.7 | 2.0 |
| Guanfacine hydrochloride | 81.1 | 0.7 | Streptomycin sulfate | 1.7 | 1.5 |
| Cinnarizine | 81.1 | 1.7 | Trimethylcolchicinic acid | 1.7 | 1.2 |
| Picotamide monohydrate | 80.9 | 35.0 | (1-[(4-Chlorophenyl)phenyl-methyl]-4-methylpiperazine) | 1.7 | 0.4 |
| Trimeprazine tartrate | 80.6 | 0.0 | Suxibuzone | 1.7 | 1.9 |
| Chlormezanone | 80.5 | 1.0 | Methyldopa (L,-) | 1.7 | 1.9 |
| Diphenidol hydrochloride | 80.5 | 2.7 | Phenethicillin potassium salt | 1.6 | 9.3 |
| Methocarbamol | 80.4 | 14.6 | Benfotiamine | 1.6 | 1.4 |
| Diethylcarbamazine citrate | 80.1 | 40.7 | Solanine alpha | 1.5 | 1.0 |
| Sulindac | 80.0 | 10.4 | Noscapine | 1.5 | 1.9 |
| Canrenoic acid potassium salt | 79.8 | 28.6 | Raloxifene hydrochloride | 1.5 | 0.4 |
| Dihydroergotamine tartrate | 79.7 | 13.3 | Carbachol | 1.5 | 0.5 |
| Fludrocortisone | 79.5 | 19.7 | Syrosingopine | 1.5 | 1.8 |
| Harmane hydrochloride | 79.3 | 25.6 | Eserine sulfate, physostigmine sulfate | 1.5 | 1.8 |
| Cyclobenzaprine hydrochloride | 79.2 | 7.1 | Benperidol | 1.4 | 1.8 |
| Methylprednisolone, 6-alpha | 78.9 | 2.1 | Colistin sulfate | 1.4 | 0.6 |
| 3-alpha-hydroxy-5-beta-androstan-17-one | 78.7 | 26.8 | Benzamil hydrochloride | 1.4 | 1.2 |
| Cholecalciferol | 78.7 | 11.2 | Drofenine hydrochloride | 1.4 | 0.9 |
| Trazodone hydrochloride | 78.5 | 15.4 | Fluorometholone | 1.3 | 1.3 |
| Ungerine nitrate | 78.4 | 17.6 | Epicatechin-(-) | 1.3 | 0.9 |
| Pyrithione sodium salt | 78.4 | 3.6 | DO 897/99 | 1.3 | 0.6 |
| Bezafibrate | 78.3 | 22.8 | Iobenguane sulfate | 1.2 | 1.5 |
| Metronidazole | 78.2 | 24.8 | Lidoflazine | 1.2 | 1.0 |
| Quinidine hydrochloride monohydrate | 78.1 | 11.8 | Mitotane | 1.2 | 0.2 |
| Roxithromycin | 78.1 | 26.9 | Betulinic acid | 1.2 | 0.5 |
| Scoulerine | 78.0 | 4.3 | Stachydrine hydrochloride | 1.1 | 1.3 |
| Dimethadione | 77.8 | 35.3 | Prochlorperazine dimaleate | 1.1 | 1.1 |
| Zidovudine, AZT | 77.7 | 6.9 | Oxethazaine | 1.1 | 0.3 |
| Sulfaquinoxaline sodium salt | 77.5 | 3.5 | Tubocurarine chloride pentahydrate (+) | 1.1 | 0.6 |
| Rolitetracycline | 77.4 | 27.2 | Flufenamic acid | 1.1 | 0.3 |
| Tiaprofenic acid | 77.3 | 6.5 | Amodiaquin dihydrochloride dihydrate | 1.1 | 0.0 |
| Morantel tartrate | 77.2 | 35.5 | Dicyclomine hydrochloride | 1.1 | 0.4 |
| Pridinol methanesulfonate salt | 77.2 | 30.2 | Retinoic acid | 1.1 | 0.7 |
| Imipramine hydrochloride | 77.0 | 21.2 | N-Acetyl-DL-homocysteine Thiolactone | 1.0 | 1.0 |
| Labetalol hydrochloride | 76.8 | 29.9 | Tolfenamic acid | 1.0 | 0.2 |
| Laudanosin, 6 -bromo (R,S) | 76.8 | 11.7 | Coralyne chloride hydrate | 1.0 | 0.6 |
| Prednisone | 76.7 | 1.5 | Fluorocurarine chloride | 1.0 | 0.2 |
| Hydroxytacrine maleate (R,S) | 76.6 | 11.4 | Chlorprothixene hydrochloride | 1.0 | 0.3 |
| Cefamandole sodium salt | 76.3 | 18.8 | Aposcopolamine | 1.0 | 0.3 |
| Bacampicillin hydrochloride | 76.1 | 6.6 | Vancomycin hydrochloride | 1.0 | 1.1 |
| Tiratricol, 3,3 ,5-triiodothyroacetic acid | 76.1 | 24.1 | Bepridil hydrochloride | 1.0 | 0.0 |
| Doxylamine succinate | 75.9 | 26.4 | Simvastatin | 0.9 | 0.4 |
| Betamethasone | 75.6 | 5.5 | Azathymine, 6 | 0.9 | 1.1 |
| Pyrimethamine | 75.5 | 41.6 | Ethaverine hydrochloride | 0.9 | 0.1 |
| Hesperetin | 75.5 | 48.1 | Artemisinin | 0.9 | 0.8 |
| Solasodine | 75.1 | 9.7 | Sevedindione | 0.9 | 0.1 |
| Nalidixic acid sodium salt hydrate | 74.8 | 12.1 | Chlorotrianisene | 0.9 | 0.2 |
| Triprolidine hydrochloride | 74.6 | 10.2 | Clemastine fumarate | 0.9 | 0.1 |
| Sparteine (-) | 73.5 | 23.4 | Lasalocid sodium salt | 0.9 | 0.3 |
| Amethopterin (R,S) | 73.5 | 2.1 | Agmatine sulfate | 0.9 | 0.7 |
| Ritodrine hydrochloride | 73.5 | 19.7 | Strophanthidin | 0.9 | 0.1 |
| Levodopa | 73.2 | 18.9 | Ethamivan | 0.9 | 0.9 |
| Diphenhydramine hydrochloride | 73.1 | 18.8 | Perphenazine | 0.9 | 0.1 |
| Mimosine | 73.0 | 17.1 | Nimesulide | 0.9 | 0.3 |
| Etifenin | 72.9 | 1.5 | Nicardipine hydrochloride | 0.9 | 0.6 |
| Vitexin | 72.4 | 7.7 | Pimozide | 0.8 | 0.2 |
| Chlorpheniramine maleate | 72.2 | 35.9 | Fluphenazine dihydrochloride | 0.8 | 0.0 |
| Streptozotocin | 71.4 | 4.1 | Strophantine octahydrate | 0.8 | 0.3 |
| Acemetacin | 71.1 | 17.2 | Lisuride (S)(-) | 0.8 | 0.2 |
| Phenformin hydrochloride | 71.1 | 2.0 | Digitoxigenin | 0.8 | 0.1 |
| Chicago sky blue 6B | 70.8 | 2.7 | Flunarizine dihydrochloride | 0.8 | 0.3 |
| Bromperidol | 70.7 | 27.4 | Diflunisal | 0.8 | 0.5 |
| Pyrantel tartrate | 70.6 | 49.2 | Pseudopelletierine hydrochloride | 0.8 | 0.8 |
| Fosfosal | 70.6 | 53.3 | Nystatine | 0.8 | 0.7 |
| Doxepin hydrochloride | 70.1 | 15.9 | Niclosamide | 0.8 | 0.4 |
| Melatonin | 70.0 | 25.1 | Loperamide hydrochloride | 0.8 | 0.4 |
| Desipramine hydrochloride | 69.9 | 15.9 | Nitrofurantoin | 0.8 | 0.4 |
| Oxybutynin chloride | 69.7 | 4.6 | Hydroquinine hydrobromide hydrate | 0.8 | 0.9 |
| Methotrexate | 69.5 | 48.5 | Pyridoxine hydrochloride | 0.8 | 0.8 |
| Arecoline hydrobromide | 69.1 | 63.1 | Terfenadine | 0.8 | 0.1 |
| Tobramycin | 68.4 | 15.3 | Disulfiram | 0.8 | 0.3 |
| Dimenhydrinate | 68.3 | 17.4 | Trifluoperazine dihydrochloride | 0.8 | 0.4 |
| Acetohexamide | 68.0 | 19.7 | Ethoxyquin | 0.8 | 0.3 |
| Hyoscyamine (L) | 67.9 | 52.6 | Meclozine dihydrochloride | 0.8 | 0.1 |
| Promazine hydrochloride | 67.8 | 10.5 | Ciclopirox ethanolamine | 0.8 | 0.4 |
| N6-methyladenosine | 67.7 | 23.7 | Menadione | 0.7 | 0.1 |
| Nalbuphine hydrochloride | 67.6 | 52.4 | Perhexiline maleate | 0.7 | 0.1 |
| Fusaric acid | 67.4 | 17.0 | Miconazole | 0.7 | 0.1 |
| Indapamide | 67.2 | 32.6 | Fenofibrate | 0.7 | 0.3 |
| Diltiazem hydrochloride | 67.1 | 46.9 | Lynestrenol | 0.7 | 0.6 |
| Aceclofenac | 67.0 | 45.2 | Methylene blue | 0.7 | 0.1 |
| Danazol | 66.9 | 56.9 | Triflupromazine hydrochloride | 0.7 | 0.1 |
| Bucladesine sodium salt | 66.9 | 52.0 | Dequalinium dichloride hydrate | 0.7 | 0.1 |
| Myricetin | 66.7 | 10.7 | Minoxidil | 0.7 | 0.2 |
| Sulfisoxazole | 66.6 | 22.3 | Doxorubicin hydrochloride | 0.7 | 0.1 |
| Azathioprine | 66.5 | 17.3 | Dihydroergocristine mesylate | 0.7 | 0.6 |
| Clindamycin hydrochloride | 66.4 | 48.3 | Bisacodyl | 0.7 | 0.2 |
| Rauwolscine hydrochloride | 66.3 | 25.9 | Proadifen hydrochloride | 0.7 | 0.1 |
| Acetylsalicylsalicylic acid | 66.3 | 44.6 | Amoxicillin | 0.7 | 0.1 |
| Harmine hydrochloride | 66.1 | 29.7 | Dienestrol | 0.7 | 2.0 |
| Pindolol | 66.0 | 54.3 | Dicumarol | 0.7 | 0.2 |
| Pirenzepine dihydrochloride | 66.0 | 56.7 | Dinoprostone | 0.7 | 0.2 |
| Cimetidine | 65.8 | 35.8 | Folinic acid calcium salt | 0.7 | 0.5 |
| Sulfabenzamide | 65.7 | 7.1 | Econazole nitrate | 0.7 | 0.4 |
| Remerine hydrochloride | 65.6 | 24.9 | Ebselen | 0.7 | 0.1 |
| Panthenol (D) | 65.3 | 56.7 | Flupentixol dihydrochloride cis-(Z) | 0.7 | 0.1 |
| Hydroxyzine dihydrochloride | 65.3 | 42.9 | Metergoline | 0.7 | 0.1 |
| Corticosterone | 63.4 | 9.6 | Sulconazole nitrate | 0.6 | 0.1 |
| Pergolide mesylate | 63.0 | 19.9 | Riboflavine | 0.6 | 0.6 |
| Sulfasalazine | 63.0 | 18.4 | Isoquinoline, 6,7-dimethoxy-1-methyl-1,2,3,4-tetrahydro, hydrochloride | 0.6 | 0.5 |
| Ciprofloxacin hydrochloride | 62.8 | 26.3 | Thioridazine hydrochloride | 0.6 | 0.3 |
| Tocopherol (R,S) | 62.6 | 80.4 | Trigonelline hydrochloride | 0.6 | 0.6 |
| Fenbendazole | 62.2 | 16.5 | Diethylstilbestrol | 0.6 | 0.1 |
| Naringin hydrate | 60.7 | 46.7 | Fendiline hydrochloride | 0.6 | 0.2 |
| Phosphocreatine di tris salt | 60.2 | 74.1 | Serotonin hydrochloride | 0.6 | 0.5 |
| Norharman | 59.9 | 46.4 | Furosemide | 0.6 | 0.0 |
| Thyroxine (L) | 59.3 | 82.5 | Suloctidil | 0.6 | 0.0 |
| Demeclocycline hydrochloride | 59.0 | 44.8 | Isotretinoin | 0.6 | 0.4 |
| Clenbuterol hydrochloride | 59.0 | 21.4 | Flutamide | 0.6 | 0.1 |
| SR-95639A | 58.4 | 80.2 | Amphotericin B | 0.6 | 0.0 |
| Brompheniramine maleate | 57.5 | 72.5 | Digoxin | 0.6 | 0.0 |
| Adamantamine fumarate | 56.8 | 79.3 | Celestin blue | 0.6 | 0.1 |
| Niridazole | 55.4 | 33.1 | Althiazide | 0.6 | 36.2 |
| Lumicolchicine gamma | 54.6 | 64.2 | Cephalothin sodium salt | 0.6 | 0.4 |
| Dehydrocholic acid | 54.4 | 6.0 | Lanatoside C | 0.6 | 0.4 |
| Methoxy-8-psoralen | 54.1 | 52.2 | Betonicine | 0.6 | 0.4 |
| Pantothenic acid calcium salt monohydrate | 53.4 | 74.8 | Clotrimazole | 0.6 | 0.1 |
| Lactobionic acid | 50.9 | 59.9 | Astemizole | 0.6 | 0.2 |
| Cisapride | 50.5 | 49.6 | Alexidine dihydrochloride | 0.6 | 0.2 |
| Estradiol-17 beta | 48.8 | 67.7 | Nifuroxazide | 0.6 | 0.4 |
| Dirithromycin | 47.8 | 63.4 | Scopolamin-N-oxide hydrobromide | 0.6 | 0.0 |
| Harmalol hydrochloride dihydrate | 47.6 | 49.4 | Clomiphene citrate (Z,E) | 0.5 | 0.1 |
| Meclocycline sulfosalicylate | 47.5 | 59.3 | Methiothepin maleate | 0.5 | 0.1 |
| Apomorphine hydrochloride (R,-) | 46.8 | 9.6 | Avermectin B1a | 0.5 | 0.1 |
| Hepes,4-(2-hydroxyethyl)-1-piperazineethanesulfonic acid | 46.7 | 56.6 | Isosorbide dinitrate | 0.5 | 0.0 |
| Harmol hydrochloride monohydrate | 46.3 | 50.4 | Mefloquine hydrochloride | 0.5 | 0.0 |
| Pimethixene maleate | 43.9 | 6.2 | Quinacrine dihydrochloride dihydrate | 0.5 | 0.2 |
| Aspartic acid, N-acetyl (R,S) | 42.0 | 51.0 | Felodipine | 0.5 | 0.1 |
| Primaquine diphosphate | 40.5 | 56.5 | Niflumic acid | 0.5 | 0.1 |
| Dinoprost trometamol | 38.2 | 42.9 | Ellipticine | 0.5 | 0.3 |
| Nortriptyline hydrochloride | 38.1 | 6.2 | Antimycin A | 0.5 | 0.2 |
| Amitryptiline hydrochloride | 36.1 | 22.1 | Aconitine | 0.5 | 0.2 |
| Anthraquinone, 1,5-diamino | 33.3 | 41.0 | Chlorhexidine | 0.5 | 0.3 |
| Apigenin | 31.4 | 41.6 | Sulmazole | 0.5 | 0.4 |
| Tolbutamide | 31.3 | 35.4 | Budesonide | 0.5 | 0.0 |
| Todralazine hydrochloride | 30.5 | 41.9 | Papaverine hydrochloride | 0.5 | 0.2 |
| Karakoline | 30.0 | 40.7 | Prenylamine lactate | 0.5 | 0.1 |
| Cyclosporin A | 29.5 | 29.5 | Berberine chloride | 0.5 | 0.2 |
| Isoetharine mesylate salt | 28.6 | 46.2 | Chrysene-1,4-quinone | 0.5 | 0.3 |
| Quercetine dihydrate | 28.5 | 29.7 | Butirosin disulfate salt | 0.5 | 0.3 |
| Androsterone | 28.4 | 39.7 | Ivermectin | 0.5 | 0.2 |
| Calciferol | 27.8 | 38.2 | Nitrofural | 0.4 | 0.4 |
| Metaproterenol sulfate, orciprenaline sulfate | 27.5 | 40.3 | Metixene hydrochloride | 0.4 | 0.2 |
| Piperlongumine | 25.1 | 21.8 | Monensin sodium salt | 0.4 | 0.4 |
| Pargyline hydrochloride | 23.9 | 17.9 | Cytisine (-) | 0.4 | 0.3 |
| Vidarabine | 23.4 | 29.1 | Merbromin | 0.4 | 0.2 |
| Mechlorethamine hydrochloride | 22.1 | 5.3 | Protoveratrine A | 0.4 | 0.2 |
| Hydralazine hydrochloride | 21.6 | 29.5 | GBR 12909 dihydrochloride | 0.4 | 0.1 |
| Semustine | 20.7 | 12.2 | Veratroylzygadenine | 0.4 | 0.3 |
| Edrophonium chloride | 20.7 | 23.0 | Cloperastine hydrochloride | 0.4 | 0.4 |
| Naloxone hydrochloride | 19.8 | 9.8 | Tamoxifen citrate | 0.4 | 0.3 |
| Delsoline | 18.4 | 17.0 | Gossypol | 0.4 | 0.4 |
| Aztreonam | 17.1 | 14.1 | Diaziquone | 0.4 | 0.2 |
| Cyproheptadine hydrochloride | 16.7 | 1.8 | Benzethonium chloride | 0.4 | 0.1 |
| Nifedipine | 15.4 | 11.5 | Methyl benzethonium chloride | 0.4 | 0.3 |
| Hemicholinium bromide | 15.2 | 9.2 | Selegiline hydrochloride | 0.4 | 0.1 |
| Chloroquine diphosphate | 15.2 | 18.6 | Sulfanilamide | 0.4 | 0.2 |
| Colchicine | 15.1 | 11.6 | Haloprogin | 0.4 | 0.2 |
| Spiramycin | 14.7 | 19.5 | Rescinnamin | 0.4 | 0.2 |
| Mebendazole | 14.6 | 10.4 | Mitoxantrone dihydrochloride | 0.4 | 0.1 |
| Albendazole | 14.4 | 6.8 | Palmatine chloride | 0.4 | 0.1 |
| Fusidic acid sodium salt | 14.3 | 18.2 | Biperiden hydrochloride | 0.3 | 0.2 |
| Camptothecine (S,+) | 14.0 | 14.9 | Thimerosal | 0.3 | 0.2 |
| Amidopyrine | 14.0 | 10.7 | Clofilium tosylate | 0.3 | 0.1 |
| Paclitaxel | 13.9 | 13.7 | Cefaclor | 0.3 | 0.2 |
| Riluzole hydrochloride | 13.5 | 14.3 | Daunorubicin hydrochloride | 0.3 | 0.0 |
| Ornidazole | 13.1 | 15.9 | Amiodarone hydrochloride | 0.2 | 0.1 |
| Verapamyl hydrochloride | 12.8 | 5.5 | Rotenone | 0.2 | 0.0 |
| Procaine hydrochloride | 12.7 | 12.7 | Gramicidin | 0.2 | 0.0 |
| Ethisterone | 12.1 | 11.4 |  |  |  |
| Clozapine | 11.8 | 5.2 |  |  |  |
| Famotidine | 11.7 | 5.7 |  |  |  |
| Luteolin | 11.6 | 1.4 |  |  |  |
| Methylhydantoin-5-(D) | 11.4 | 0.0 |  |  |  |
| Moroxidine hydrochloride | 11.3 | 12.2 |  |  |  |
| Nocodazole | 11.3 | 8.5 |  |  |  |
| Dipyridamole | 11.1 | 9.1 |  |  |  |
| Gallamine triethiodide | 10.9 | 5.7 |  |  |  |
